# Preclinical study of engineering MSCs promoting diabetic wound healing and other inflammatory diseases through M2 polarization

**DOI:** 10.1186/s13287-025-04248-y

**Published:** 2025-03-05

**Authors:** Di Wu, Rencun Liu, Xiaotong Cen, Wanwen Dong, Qing Chen, Jiali Lin, Xia Wang, Yixia Ling, Rui Mao, Haitao Sun, Rui Huang, Huanxing Su, Hongjie Xu, Dajiang Qin

**Affiliations:** 1 Key Laboratory of Biological Targeting Diagnosis, Therapy and Rehabilitation of Guangdong Higher Education Institutes, The Fifth Affiliated Hospital, Guangzhou Medical University, Guangzhou, China; 2 Shandong Province Key Laboratory of Detection Technology for Tumour Makers, School of Chemistry and Chemical Engineering, Linyi University, Linyi, China; 3 State Key Laboratory of Ophthalmology, Guangdong Provincial Key Laboratory of Ophthalmology and Visual Science, Zhongshan Ophthalmic Center, Sun Yat-Sen University, Guangzhou, China; 4 Bioland Laboratory, Guangzhou Regenerative Medicine and Health Guangdong Laboratory, Guangzhou, China; 5 Laboratory Animal Research Center, Guangzhou Institutes of Biomedicine and Health, Chinese Academy of Sciences, Guangzhou, China; 6 Neurosurgery Centre, Department of Cerebrovascular Surgery, Engineering Technology Research Centre of Education Ministry of China on Diagnosis and Treatment of Cerebrovascular Disease, Guangdong Provincial Key Laboratory on Brain Function Repair and Regeneration, The Neurosurgery Institute of Guangdong Province, Zhujiang Hospital, The National Key Clinical Specialty, Southern Medical University, Guangzhou, Guangdong China; 7 The Fifth Clinical College, Guangzhou Medical University, Guangzhou, China; 8 State Key Laboratory of Quality Research in Chinese Medicine, Institute of Chinese Medical Sciences, University of Macau, Macao, China; 9 Centre for Regenerative Medicine and Health, Hong Kong Institute of Science & Innovation, Chinese Academy of Sciences, Hong Kong SAR, China; 10 No.621 Gangwan Road, Huangpu District, Guangzhou, China

**Keywords:** Mesenchymal stem cells, Macrophage, Diabetic wound healing, Posttraumatic inflammation, Tissue repair, Regeneration

## Abstract

**Background:**

Diabetic foot ulcer (DFU) represents a common and severe complication of diabetes mellitus. Effective and safe treatments need to be developed. Mesenchymal stem cells (MSCs) have demonstrated crucial roles in tissue regeneration, wound repair and inflammation regulation. However, the function is limited. The safety and efficacy of gene-modified MSCs is unknown. Therefore, this study aimed to investigate whether genetically modified MSCs with highly efficient expression of anti-inflammatory factors promote diabetic wound repair by regulating macrophage phenotype transition. This may provide a new approach to treating diabetic wound healing.

**Methods:**

In this study, human umbilical cord-derived MSCs (hUMSCs) were genetically modified using recombinant lentiviral vectors to simultaneously overexpress three anti-inflammatory factors, interleukin (IL)-4, IL-10, IL-13 (MSCs-3IL). Cell counting kit-8, flow cytometry and differentiation assay were used to detect the criteria of MSCs. Overexpression efficiency was evaluated using flow cytometry, quantitative real-time PCR, Western blot, enzyme-linked immunosorbent assay, and cell scratch assay. We also assessed MSCs-3IL’s ability to modulate Raw264.7 macrophage phenotype using flow cytometry and quantitative real-time PCR. In addition, we evaluated diabetic wound healing through healing rate calculation, HE staining, Masson staining, and immunohistochemical analysis of PCNA, F4/80, CD31, CD86, CD206, IL-4, IL-10 and IL-13. In addition, we evaluated the safety of the MSCs-3IL cells and the effect of the cells on several other models of inflammation.

**Results:**

MSCs-3IL efficiently expressed high levels of IL-4 and IL-10 (mRNA transcription increased by 15,000-fold and 800,000-fold, protein secretion 400 and 200 ng/mL), and IL-13 (mRNA transcription increased by 950,000-fold, protein secretion 6 ng/mL). MSCs-3IL effectively induced phenotypic polarization of pro-inflammatory M1-like macrophages (M1) towards anti-inflammatory M2-like macrophages (M2). The enhancement of function does not change the cell phenotype. The dynamic distribution *in vivo* was normal and no karyotype variation and tumor risk was observed. In a mouse diabetic wound model, MSCs-3IL promoted diabetic wound healing with a wound closure rate exceeding 96% after 14 days of cell treatment. The healing process was aided by altering macrophage phenotype (reduced CD86 and increased CD206 expression) and accelerating re-epithelialization.

**Conclusions:**

In summary, our study demonstrates that genetically modified hUMSCs effectively overexpressed three key anti-inflammatory factors (IL-4, IL-10, IL-13). MSCs-3IL-based therapy enhances diabetic wound healing with high efficiency and safety. This suggests that genetically modified hUMSCs could be used as a novel therapeutic approach for DFU repair.

**Supplementary Information:**

The online version contains supplementary material available at 10.1186/s13287-025-04248-y.

## Introduction

Diabetes mellitus (DM) is a widespread chronic condition. According to the International Diabetes Federation, 540 million adults aged 20–79 years worldwide were diagnosed with diabetes in 2024 [1]. This number is projected to rise to 643 million by 2030 and 783 million by 2045 [2–4]. Diabetic foot ulcer (DFU) is a serious and prevalent complication among diabetic patients, potentially resulting in amputation in severe cases [5, 6]. Wound healing is a complex and tightly regulated biological process divided into four stages:hemostasis, inflammation, proliferation and remodeling [7]. Studies have identified diabetic neuropathy, peripheral vasculopathy, and inflammation as primary factors contributing to DFU progression, potentially stalling healing at any stage of wound healing [8, 9]. Despite traditional therapies such as debridement, dressings, antibiotics, hyperbaric oxygen therapy, negative pressure therapy, and surgery, achieving complete DFU healing remains challenging [10–12].

In the last years, cell therapy has gradually come into the limelight as a therapeutic possibility [13, 14]. In particular, mesenchymal stem cells (MSCs), as a kind of pluripotent stem cells with high self-renewal and multidirectional differentiation potential, can differentiate into osteoblasts, chondrocytes, and chondrogenic cells under specific conditions [15]. Human umbilical cord-derived MSCs (hUMSCs) have greater expansion capacity and are free from ethical constraints [16]. MSCs have been used in a variety of disease models, such as cancer, diabetes, neurological disorders, cardiovascular and pulmonary diseases [17–19]. MSCs repair damaged tissues by paracrine cytokines, which reduce inflammation, eliminate fibrosis, and promote cellular proliferation, leading to the promotion of wound healing and repair of tissues and organs [20]. However, the innate function of MSCs is not always sufficient for therapeutic use [21]. Genetic modification significantly enhances the biological functions of MSCs and circumvents certain limitations of MSCs-based repair therapy [22–24]. Therefore, using genetically engineered MSCs for diabetic wound tissue repair is imperative [25, 26].

As essential components of the innate immune system and cellular immunity, macrophages maintain tissue homeostasis and play indispensable roles throughout wound healing [27, 28]. Macrophages are classified into proinflammatory M1-like macrophages (M1) and anti-inflammatory M2-like macrophages (M2) [29]. M2 macrophages promote angiogenesis and fibroblast proliferation, migration, and differentiation, which are necessary for tissue repair. Interleukin-4 (IL-4) and interleukin-13 (IL-13) polarize M2 macrophages to reduce inflammation [30]. Increasing the M2 phenotype and decreasing the M1 phenotype have been shown to facilitate diabetic wound repair [31]. MSCs predominantly modulate the immune system via paracrine signaling, secreting cytokines such as the Th2 cytokines interleukin-10 (IL-10) and IL-4, which polarize macrophages from the M1 to the M2 phenotype, thereby reducing inflammation [29, 32]. Cytokines are signaling molecules of the immune system that regulate fundamental biological processes such as host defense, inflammation, cell growth, angiogenesis, and tissue repair [33]. During skin wound healing, IL-4 and IL-13 promote fibroblast chemotaxis, proliferation, collagen and ECM production [34, 35]. IL-10 is an anti-inflammatory and antifibrotic cytokine that promotes scarless regenerative wound healing [36, 37].

Genetic engineering may endow MSCs with greater regenerative capacity, thereby overcoming the clinical challenges of MSCs-based treatment of diabetic wounds. Previous genetic modifications of cells have focused mostly on a single specific cytokine, growth factor, or chemokine, whereas tissue regeneration requires multiple interactions with cytokines, growth factors, or chemokines [38]. Compared with MSCs overexpressing IL-4 alone, those co-overexpressing with PDGF-BB and IL-4 have been shown to increase cell proliferation, activity, and osteogenic capacity [39]. Therefore, we hypothesised that co-modification of MSCs with cytokines (MSCs-3IL) is a promising therapeutic strategy for diabetic foot ulcer repair.

**Fig. 1:**
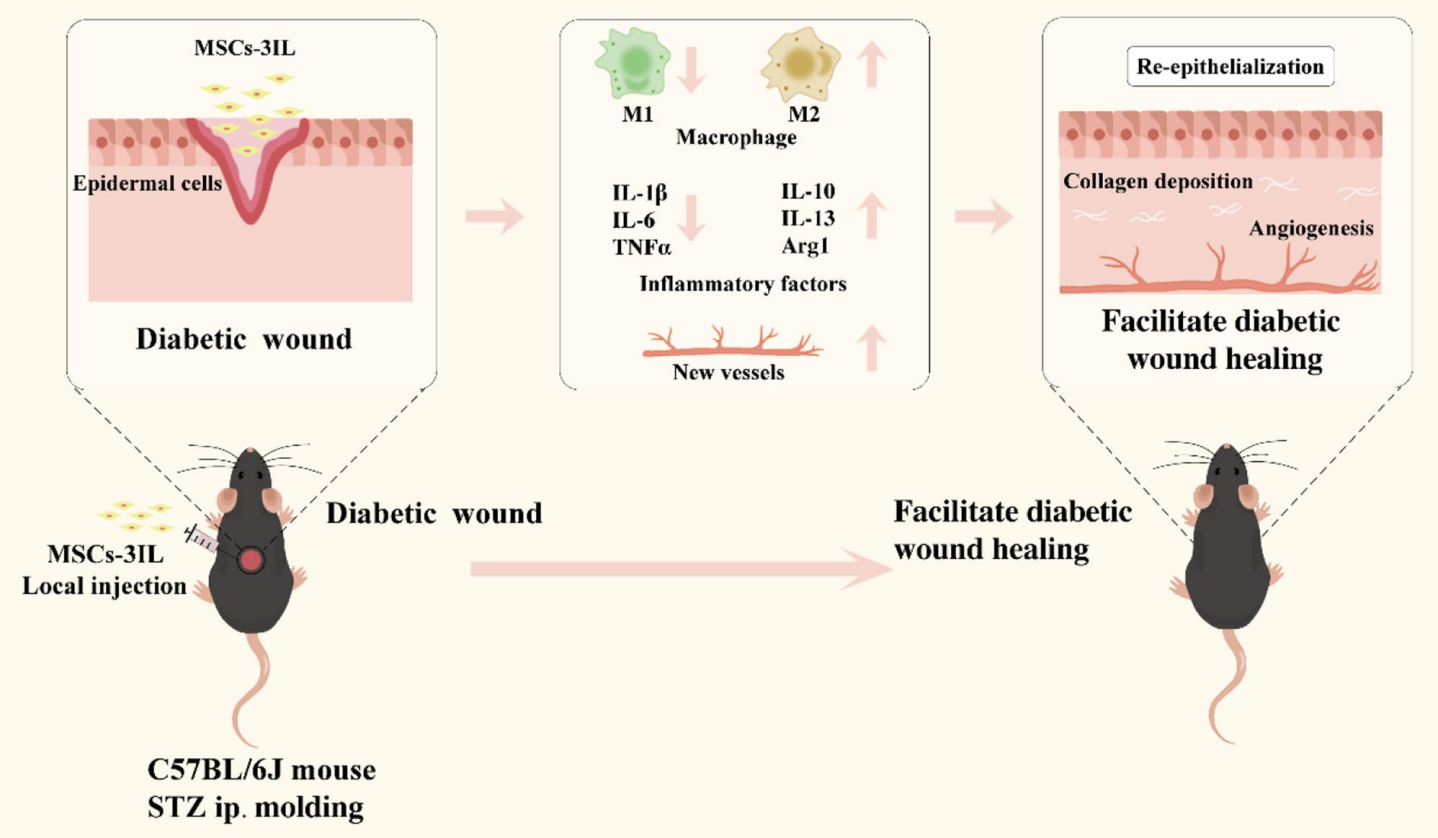
Schematic illustrations of MSCs-3IL for diabetic wound healing and closure process. C57BL/6J mice were injected with streptozotocin (STZ) to create a diabetic model, and MSCs-3IL were injected into the full-thickness skin wound, which increased the expression of anti-inflammatory factors and decreased the expression of pro-inflammatory factors. MSCs-3IL induced the polarization of M1 to M2 macrophages, promoted collagen regeneration, angiogenesis and re-epithelialization, thereby facilitating wound healing

In this study, we employed recombinant lentiviral vectors to modify hUMSCs by overexpressing three anti-inflammatory factors, namely, IL-4, IL-10, and IL-13 (MSCs-3IL). We examined the expression of these factors in MSCs-3IL and the effect of MSCs-3IL on macrophage polarization in vitro. Additionally, using a full-thickness skin wound model in diabetic mice, we investigated whether MSCs-3IL could enhance wound repair by modulating the macrophage phenotype (Fig. 1). The potential of MSCs-3IL for the treatment of DFU and safety issues were evaluated.

## Methods

### Animals and ethics approval statement

All procedures related to the animal experiments were performed in accordance with the Guide for the Care and Use of Laboratory Animals (NIH Publication No. 80 − 23). All animal studies and hUMSCs isolation and use were conducted with approval (No. IACUC-2022056, No. IACUC-2024099 and N0. KY01-2023-01-01). C57BL/6J mice (6–8 weeks old, 25 g, male), NOD-SCID immunodeficient mice (6–8 weeks old, 20 g, female) and SD rats (6–8 weeks old, 20 g, female) were obtained from GemPharmatech (Guangdong, China). All mice were maintained at controlled temperature (24 °C ± 1 °C) and relative humidity (50–60%) with a 12 hours (h) light/12 h dark light cycle. All mice were allowed free access to food and water. All efforts were made to minimize animal usage and suffering. The work has been reported in line with the ARRIVE guidelines 2.0.

### Isolation, culture and identification of hUMSCs

Umbilical cords (approximately 1.6 cm in diameter) were obtained from healthy full-term cesarean-delivered fetuses. The amniotic membrane was removed to obtain Wharton’s jelly. Wharton’s jelly was dissected into 2 mm^3^ tissue blocks and cultured with Dulbecco’s modified Eagle’s medium/F12 (DMEM/F12, VivaCell, Germany) supplemented with 10% fetal bovine serum (FBS, VivaCell, Germany) and 1% penicillin/streptomycin (P/S, HyClone, USA) at 37 °C with 5% CO_2_. The cells that migrated from the tissue blocks were considered first-passage MSCs (P0). Upon reaching approximately 80% confluency, the cells were detached with 0.25% trypsin solution (Gibco, USA), collected, and passaged or frozen. Cells from passages 4 to 8 (P4–P8) were chosen for further experiments. The expression of MSCs-positive markers (CD105, CD73, and CD90) or MSCs-negative markers (CD14, CD19, HLA-DR, CD34, and CD45) (BD Biosciences, USA) was confirmed via flow cytometry (LSRFortessa X20, BD). The multilineage differentiation potential of hUMSCs was assessed by culture in osteogenic, adipogenic, and chondrogenic differentiation media (OriCell, NZ), followed by staining with alizarin red S, oil red O, and alcian blue.

### Construction and characterization of IL-4-, IL-10-, and IL-13-modified hUMSCs

The lentiviral vectors were obtained from VectorBuilder, Inc. Vector maps are provided in Supplementary Fig. 1A (Figure S1A). hUMSCs (1.2 × 10^5^/well) were seeded in six-well plates, and the virus was diluted in MSCs culture medium supplemented with 8 µg/mL polybrene and infected with hUMSCs at a multiplicity of infection (MOI) of 20. Three groups were included in this study: (1) MSCs, unaltered MSCs; (2) MSCs-V, MSCs infected with the empty lentiviral vector; and (3) MSCs-3IL, MSCs infected simultaneously producing IL-4, IL-10 and IL-13 under a strong promoter, containing enhanced green fluorescent protein (eGFP) reporters. The cells were observed under a fluorescence microscope 48 h after infection. The infection efficiency and expression of the MSCs markers CD105, CD73, CD90, CD14, HLA-DR, and CD34 were evaluated via fluorescence microscopy and flow cytometry. Infected MSCs were cultured in osteogenic, adipogenic, and chondrogenic differentiation media to assess potential impacts on multilineage differentiation ability.

### Karyotype analysis

Karyotype analysis was performed to assess whether viral infection altered the morphological structure and number of chromosomes of hUMSCs. The cells were treated with colchicine (0.2 µg/mL) for 2.5 h at 70% confluency, followed by collection and treatment with 0.075 mol/L KCl in a hypotonic solution. Preparations were fixed, G-banding was performed, and cells in the division phase were photographed and analyzed.

### Cell proliferation assay

The cell proliferation capacity was assessed via a cell counting kit-8 (CCK-8) assay (Beyotime, China). MSCs, MSCs-V and MSCs-3IL were seeded into 96-well plates (3 × 10^3^/well, 100 µL). For measurement, 10 µL of CCK-8 solution was added to each well. After incubation for 3 h, the absorbance was measured at 450 nm via a microplate reader (MultiskanSKY, Thermo).

### Quantitative real-time PCR (qRT‒PCR) analysis

Total RNA was isolated from cells via TRIzol reagent (Invitrogen, USA), followed by reverse transcription into cDNA via the HisScript cDNA Synthesis Kit (Vazyme, China). Then, qRT‒PCR was performed via ChamQ SYBR qPCR Master Mix (Vazyme, China) on a CFX96 real-time qPCR instrument (Bio‒Rad, USA). The relative expression levels of the target genes were calculated via the 2^−ΔΔCt^ method. GAPDH and β-actin were used as internal controls. The sequences of primers used are listed in Table 1.

**Table 1:** Sequences of primers used for qRT-PCR. Human(h)-specific primers were designed to detect the expression of genes in MSCs-3IL, and mouse(m)-specific primers were designed to detect the expression of inflammatory and anti-inflammatory factors of Raw264.7

Genes	Forward primer 5′→3′	Reverse primer 3′→5′
hGAPDH	CGATATGGACGTACACACGA	TGGGTTAGCTGATAGGAGAG
h-IL-4	ATGGGTCTCACCTCCCAACT	GATGTCTGTTACGGTCAACTCG
h-IL-10	TCAAGGCGCATGTGAACTCC	GATGTCAAACTCACTCATGGCT
h-IL-13	ACGGTCATTGCTCTCACTTGCC	CTGTCAGGTTGATGCTCCATACC
mβ-actin	AAATCGTGCGTGACATCAAAGA	GCCATCTCCTGCTCGAAGTC
m-IL-1β	ACCTTCCAGGATGAGGACATGA	CTAATGGGAACGTCACACACCA
m-TNFα	GCCACCACGCTCTTCTGTCTAC	GGGTCTGGGCCATAGAACTGAT
m-IL-10	CTTACTGACTGGCATGAGGATCA	GCAGCTCTAGGAGCATGTGG
m-IL-13	CAGCCTCCCCGATACCAAAAT	GCGAAACAGTTGCTTTGTGTAG
m-IL-6	TCTATACCACTTCACAAGTCGG	GAATTGCCATTGCACAACTCTT
m-Arg1	TGGCTTTAACCTTGGCTTGCTTCG	AAAGAACAAGCCCTTGGGAGGAGA

### Enzyme-linked immunosorbent assay (ELISA)

The expression levels of IL-4, IL-10 and IL-13 in MSCs-3IL were quantitated via ELISA kits (Bluegene, China). The cell culture supernatants were collected after 72 h, and the optical densities were determined at 450 nm via a microplate reader.

### Western blotting

The total protein content was quantified via a BCA assay (Thermo, USA). Proteins were separated by sodium dodecyl sulfate‒polyacrylamide gel electrophoresis (SDS‒PAGE) and transferred to polyvinylidene fluoride (PVDF) membranes. Primary antibodies, including anti-IL-4 (Proteintech, 1:1000, USA), anti-IL-10 (RD, 1:2000, USA), and anti-IL-13 (Abcam, 1:1000, UK), were incubated with the samples overnight at 4 °C. The membranes were incubated with appropriately diluted secondary antibodies (anti-mouse IgG or anti-rabbit IgG), followed by color development and quantification.

### Cell scratch assay

MSCs, MSCs-V and MSCs-3IL cells were first pretreated with 10 µg/mL mitomycin C for 2 h to inhibit the proliferative ability of cells. Then the cells were inoculated in 96-well plates at a density of 2.5 × 10^4^/well, with 6 replicate wells in each group. After 24 h of inoculation, the cells were scratched at a density of 90% or more. After scratching, the cell culture was photographed and recorded in the Incucyte S3 instrument until the top and bottom of the cell scratches were in contact with each other. The cell migration rate was calculated as [(initial scratch distance - scratch distance at a time point)/initial scratch distance]×100%.

### Agarose spot assay

0.1 g agarose was added to 20 mL of DPBS to make a 0.5% agarose solution. The solution was heated to boiling, filtered through 0.22 μm, and cooled to 40 °C. The solution was then analyzed in an EP tube. Prepare 10 µL of PBS, IL-1β (concentration of 20 ng/mL) in EP tubes, and add 90 µL of agarose solution to mix well. Pipette 5 µL of the mixture into a 6-well plate, 8 drops per well. The 6-well plate was cooled at 4 ℃ for 15 min. Add cells (MSCs, MSCs-V, MSC-3IL) pre-treated with 10 µg/mL of mitomycin C for 2 h at a density of 5 × 10^5^/well, and add culture medium DMEM/F12 + 10% FBS + P/S. The cells were cultured in an incubator and Image J analyzed the distance of cell penetration through agarose.

### In vitro inflammation model

Raw264.7 (murine monocyte macrophage) cells were purchased from the Guangzhou Institutes of Biomedicine and Health, Chinese Academy of Sciences (Guangzhou, China). Raw264.7 cells were cultured with DMEM/HG (Gibco, USA) supplemented with 10% FBS (Gibco, USA) at 37 °C with 5% CO_2_.

Raw264.7 cells were seeded at 3 × 10^5^/well in a six-well plate and treated with or without 100 ng/mL lipopolysaccharide (LPS, Sigma, USA) for 24 h. Then, the cells were collected after 72 h of coculture with MSCs or MSCs-3IL supernatants. The impact of MSCs or the MSCs-3IL supernatant on Raw264.7 phenotypic polarization was assessed via qRT‒PCR for the expression of the inflammatory and anti-inflammatory factors *IL-1β*, *TNFα*, *IL-10*, *IL-13*, *IL-6*, *and Arg1* and via flow cytometry for CD80 and CD163 expression.

### In vivo luciferase imaging in mice

D-Luciferin (Macklin, China) was diluted to 15 mg/mL in DPBS and sterile-filtered through a 0.22-micron filter. hUMSCs were infected with a virus carrying the luciferase (Luc)-encoding gene. Untreated cells and infected cells were injected intraperitoneally (i.p.) into mice with 5 × 10^6^/100 µL DPBS, and the control group was injected with an equal volume of DPBS. At 0, 3, and 7 days after cell injection, the mice were placed in an isoflurane induction chamber, and 0.2 mL of luciferin (20 mg/mL) was injected i.p. after the animal was sedated. One group of mice was a sham-operated group, in which the mice were injected with fluorescein only and received no prior treatment. The luciferin substrate was injected 10–20 min before image capture.

### Tumorigenicity tests

To assess the safety of MSCs-3IL *in vivo*, 1 × 10^6^ cells in PBS containing 50% Matrigel were injected subcutaneously (i.h.) into NOG-SCID mice(6–8 weeks old, female). Equal volumes of DPBS were used as a negative control, and equal volumes of human induced pluripotent stem cells (hiPSCs) were used as a positive control. hiPSCs were cultured in Matrigel-coated dishes with mTeSR1 cell culture medium (StemCell, CA). Tumor formation was assessed 6 weeks after cell injection. Heart, liver, spleen, lung and kidney tissues were collected for HE staining.

### Modeling and cell transplantation in diabetic mice

C57BL/6J male mice were fasted for 16 h before treatment. Immediately before injection, streptozotocin (STZ) was dissolved in sodium citrate buffer (pH 4.5) to a final concentration of 20 mg/mL. STZ was injected i.p. into the animals at 150 mg/kg. Equal volumes of citrate buffer were used as a control for the healthy group. Diabetic mice were generated if fasting blood glucose measurements were ≥ 16.7 mmol/L on day 10. The mice were anesthetized with 0.2 mL/10 g of tribromoethanol (Sigma, Germany). Two circular, full-thickness skin wounds 8 mm in diameter were created on the backs of the mice, and cells were injected into the full-thickness skin wounds. The mice were divided into groups receiving PBS (injected with 100 µL of PBS), MSCs-V (injected with 5 × 10^4^ MSCs-V cells in 100 µL of PBS), MSCs-3IL (injected with 5 × 10^4^ MSCs-3IL cells in 100 µL of PBS), or MSCs-3IL-3d (injected with 5 × 10^4^ MSCs-3IL cells in 100 µL of PBS 3 days after wound surgery), with 25 mice per group. Wound healing was assessed via ImageJ software at 0, 3, 7, and 14 days (D0, D3, D7 D14). The wound healing rate was calculated as [(C_0_-Ct)/C_0_]×100%. C_0_ represents the wound size on day 0, and Ct represents the wound size at each time point.

### Histology and immunohistochemistry

All mice were euthanised by cervical dislocation. The skin tissues were harvested from the mice on D0, D3, and D7 D14, fixed in 4% formaldehyde, dehydrated through a gradient and then embedded in paraffin. Histological analyses included hematoxylin-eosin (HE) staining, Masson’s trichrome staining, and immunohistochemistry (PCNA, F4/80, CD31, CD86, and CD206; IL-4. IL-10 and IL-13) (Abcam, UK). Image J was used to calculate the mean value of the positive area percentage for immunohistochemical staining. Positive area percentage was calculated as (positive area/total area) ×100%.

### Inflammation modeling and cell therapy

In the vaginitis model, female rats were divided into four groups: normal control, model, MSCs-V-treated, and MSCs-3IL-treated, five per group. Except for the normal control group, 0.2 mL of 25% phenol glue paste (composed of 5 mL of liquid phenol, 1 g of gum arabic powder, 4 mL of glycerol, and distilled water to a total volume of 20 mL) was injected approximately 1 cm deep into the vagina once daily for a total of four injections. Following the fourth model, 1 × 10^5^/50 µL of MSCs-V were injected into the vaginas of the rats in the MSCs-V treatment group, an equivalent volume of MSCs-3IL was injected into the MSCs-3IL treatment group, and 50 µL of PBS was injected into the model group. Seven days after cell therapy, the rats were euthanized, and vaginal samples were collected for HE staining.

In the rat osteoarthritis (OA) model, the rats were allocated to four groups: normal control, model, MSCs-V-treated, and MSCs-3IL-treated, five per group. OA was induced in rats via meniscus injury surgery. Four weeks postsurgery, 1 × 10^6^/50 µL of MSCs-V were injected into the joint cavities of the rats in the MSCs-V treatment group, an equivalent volume of MSCs-3IL was administered to the MSCs-3IL treatment group, and 50 µL of PBS was injected into the model group. At week 10, the rats were euthanized, and knee joint tissues were collected for HE and Masson staining.

In the mouse acute pleurisy model, the mice were allocated to four groups: normal control, model, MSCs-V-treated, and MSCs-3IL-treated, five per group. Each mouse received an injection of 30 µL of 1% carrageenan into the right pleural cavity, while the normal control group was administered an equivalent volume of saline. Two hours later, 5 × 10^5^/50 µL MSCs-V were injected into the right pleural cavity of the MSCs-V treatment group, and an equivalent volume of MSCs-3IL was administered to the MSCs-3IL treatment group. The mice were euthanized 6 h after cell therapy, and the lungs were collected for HE staining.

For the mouse acute inflammation model, the mice were allocated to four groups: normal control, model, MSCs-V-treated, and MSCs-3IL-treated, five per group. All the groups, except for the normal control group, which received an intraperitoneal injection of saline, were injected with 100 µL of a 6 mg/mL LPS solution to induce acute inflammation. Two hours after model induction, the MSCs-V treatment group was administered an intraperitoneal injection of 1 × 10^6^/200 µL of MSCs-V cells, the MSCs-3IL treatment group received an equivalent volume of MSCs-3IL cells, and the model group was administered 200 µL of PBS. The mice were euthanized 6 hours after cell therapy, and the right lung and spleen were collected for HE staining.

For the acute colitis mouse model, the mice were divided into four groups: normal control, model, MSCs-V-treated, and MSCs-3IL-treated. The mice in the normal control group were given distilled water ad libitum, while those in the remaining groups were provided 2% dextran sulfate sodium (DSS) for 5 days. On the third day of induction, the MSCs-V and MSCs-3IL treatment groups were administered an intraperitoneal injection of 5 × 10^5^ cells in 200 µL, whereas the model group was administered an equivalent volume of saline. On the seventh day after induction, all the mice were euthanized by cervical dislocation, and the distal colon were collected for HE staining.

To establish a diabetic mouse model, LPS (5 mg/kg) was injected intraperitoneally to establish a model of diabetes complicating pneumonia. The diabetic mice were divided into 4 groups, namely, the normal control group, model group, MSCs-V treatment group and MSCs-3IL treatment group, and the cells were injected intraperitoneally at 5 × 10^5^/200 µL. The mice were cervically dislocated and killed 6 h after injection, and the right lungs were harvested and stained with HE.

### Statistical analysis

All the data were analyzed with GraphPad Prism 9 and reported as the means ± standard deviations. One-way ANOVA with multiple comparisons was used to compare the significant differences. *P value < 0.05 was considered statistically significant. The article used ChatGPT to embellish and syntactically modify the statements.

## Results

### Characterization of hUMSCs

The primary hUMSCs were identified by their morphological characteristics, surface marker identification, and multidirectional differentiation potential. Most cells exhibited fibroblast-like adherent growth under the microscope, with some appearing spindle shaped or irregularly triangular (Figure S1B). The expression of cell surface markers was identified by flow cytometry, which revealed > 95% positivity for CD105, CD73, CD90, and CD105 but negativity for CD34, CD45, CD14, and HLA-DR < 2% (Figure S1C). Alizarin red staining revealed that the cells were able to differentiate into osteoblasts, oil red O staining revealed that the cells differentiated into adipocytes, and alcian blue staining revealed that they differentiated into chondrocytes (Figure S1D), confirming their multipotent characteristics. These results indicate that the hUMSCs we isolated have stem cell characteristics.

### Viral infection did not alter the characteristics of hUMSCs

**Fig. 2:**
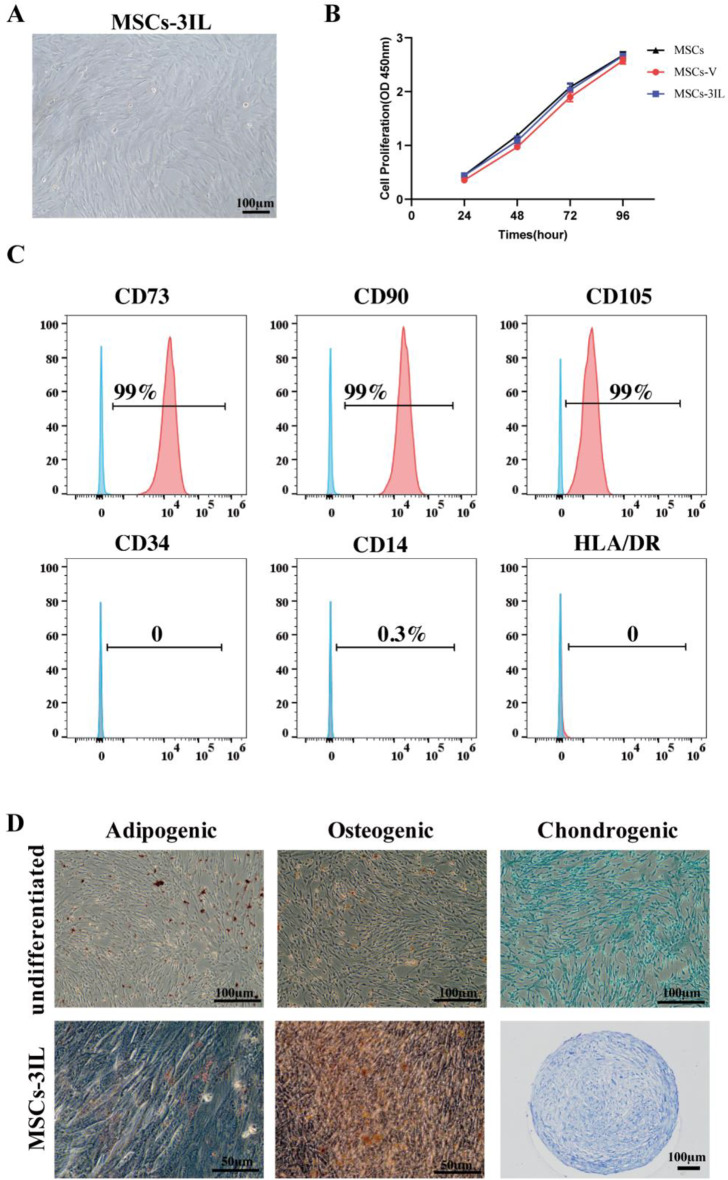
Characterization of MSCs-3IL. (**A**) Microscopic image of cultured MSCs-3IL showing normal morphology, scale bar:100 μm. (**B**) Cell proliferation was assessed by CCK-8 assay. (**C**) Flow cytometry analysis of MSCs-3IL surface markers: CD73, CD90, CD105 (positive markers); CD34, CD14, HLA-DR (negative markers). (**D**) Differentiation potential of MSCs-3IL demonstrated by adipogenic (oil red O staining), osteogenic (alizarin red S staining), and chondrogenic (alizarin blue staining) differentiation compared to undifferentiated control MSCs, scale bar: 50 μm, 100 μm

To enhance therapeutic efficacy, hUMSCs were genetically modified to overexpress IL-4, IL-10, and IL-13 simultaneously. Analysis of cell morphology, stem cell phenotype, and differentiation potential revealed no alterations in biological properties due to viral infection. The cultured MSCs-3IL were spindle shaped (Fig. 2A). A CCK-8 assay was performed to determine the proliferation of the cells after 1, 2, 3 and 4 days. The proliferation capacity of virus-infected cells was comparable to that of untreated cells (Fig. 2B). The levels of the stem cell surface markers CD105, CD73, CD90 and CD105 were > 95%, whereas those of CD34, CD45, CD14 and HLA-DR were < 2% (Fig. 2C). Alizarin red, oil red O, and alcian blue staining revealed osteogenic, adipogenic, and chondrogenic differentiation, respectively (Fig. 2D). The chromosomes of the modified cells were not altered, as shown by karyotypic analysis (Figure S1E). These data indicate that viral infection does not alter the characteristics of hUMSCs.

To assess postinfection safety, hUMSCs were infected with a luciferase-expressing virus. *In vivo*, imaging with D-Luciferin detected infected hUMSCs on D0 and D3, and weak fluorescence was detected on D7 (Figure S2A). This indicates that these bacteria can survive *in vivo* without the safety concerns of prolonged presence. Additionally, when MSCs-3IL were transplanted into NOD-SCID mice, samples were taken 6 weeks after injection, and no adverse effects on proliferation, tumorigenicity, or other safety concerns were observed in the MSCs-3IL group. Tumor formation at the injection site was observed in the positive control hiPSC group at 4 weeks (Figure S2B). HE staining of the heart, liver, spleen, lung and kidney revealed no significant differences between the MSCs-3IL and DPBS groups (Figure S2C). These data confirmed the safety profile of the lentiviral-modified hUMSCs.

### Modified hUMSCs efficiently overexpress IL-4, IL-10 and IL-13

**Fig. 3:**
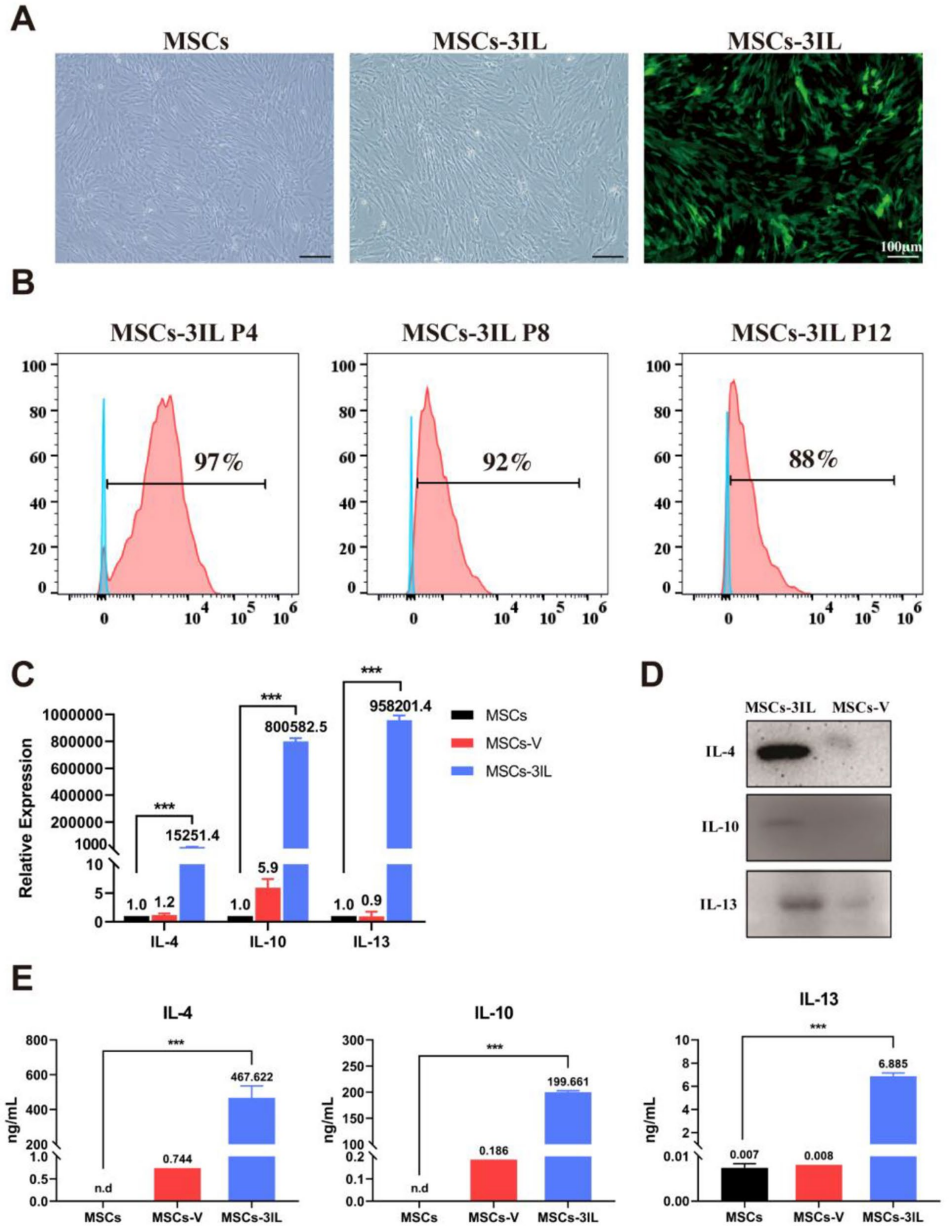
Expression level of IL-4, IL-10, and IL-13 in MSCs-3IL. (**A**) Fluorescence microscopy image showing MSCs-3IL cells expressing green fluorescent protein (eGFP), scale bar: 100 μm. (**B**) Flow cytometric analysis demonstrating the infection efficiency of MSCs-3IL at different passages. (**C**) QRT-PCR analysis of *IL-4*, *IL-10*, and *IL-13* gene expression in MSCs-3IL compared to control MSCs. Statistical analysis was performed using one-way ANOVA. ***P < 0.001 compared to the control group. *n* = 3 biological replicates. Error bars represent standard deviations (SDs). (**D**) Western blot analysis showing protein expression levels of IL-4, IL-10, and IL-13 in MSCs-3IL. (**E**) ELISA results measuring IL-4, IL-10, and IL-13 secretion levels in MSCs-3IL cell supernatants collected after 72 h of culture. n.d: below the detectable range of ELISA. ***P < 0.001 compared to the control group. *n* = 3 biological replicates. Error bars indicate standard deviations (SDs)

To investigate the infection efficiency and expression levels of IL-4, IL-10, and IL-13 in modified MSCs, we examined their fluorescence and secretion levels. Following 48 h of lentiviral infection, MSCs-3IL cells expressed green fluorescence (eGFP) under a fluorescence microscope (Fig. 3A). Flow cytometry analysis revealed an infection rate of 97% for eGFP expression, with a minimal decrease observed after multiple passages (92% and 88%), indicating high virus infection efficiency (Fig. 3B). qRT‒PCR analysis (Fig. 3C) revealed that MSCs-3IL expressed *IL-4*, *IL-10*, and *IL-13* at millions of times higher levels than MSCs and MSCs-V cells. Western blotting confirmed elevated protein levels of IL-4, IL-10, and IL-13 in the MSCs-3IL group compared with those in the MSCs-V group (Fig. 3D). IL-4, IL-10 and IL-13 secretion levels in the culture medium were assayed via ELISA. IL-4 and IL-10 secretion in the MSCs group was below the detectable range of ELISA (n.d.). IL-4, IL-10 and IL-13 levels were greater in the MSCs-3IL group than in the MSCs and MSCs-V groups (Fig. 3E). MSCs-3IL efficiently expressed high levels of IL-4 (protein secretion > 400 ng/mL), IL-10 (protein secretion 200 ng/mL) and IL-13 (6 ng/mL), which significantly enhanced cell secretion. These findings highlight the efficient viral infection and robust expression of IL-4, IL-10, and IL-13 by modified hUMSCs.

### MSCs-3IL polarized LPS-stimulated macrophages to the M2 phenotype

**Fig. 4:**
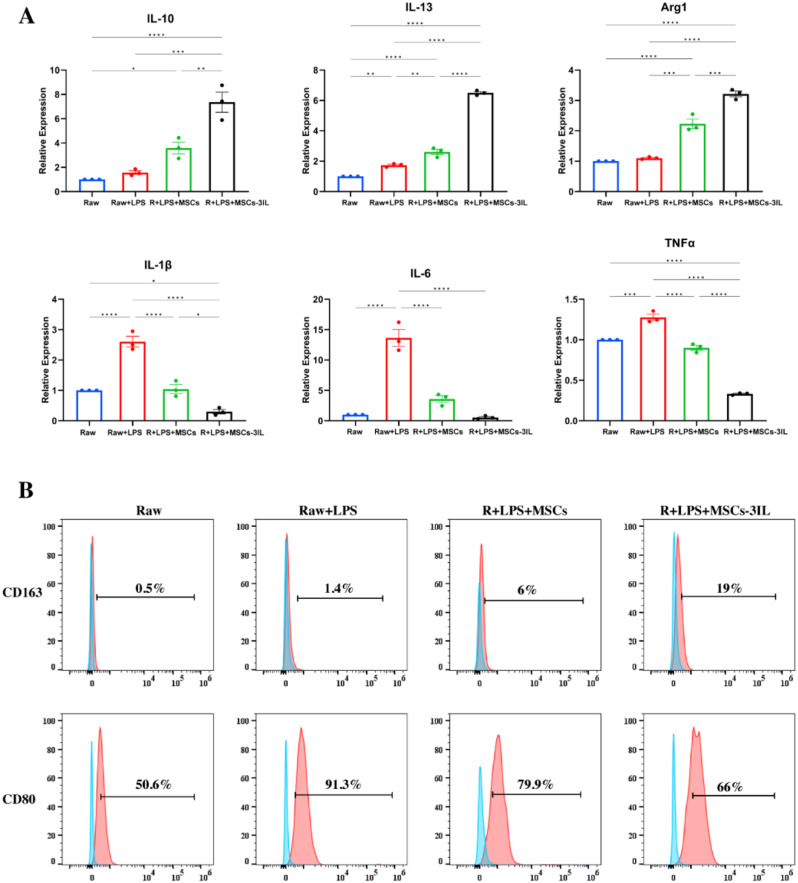
MSCs-3IL suppressed the inflammatory reaction and enhanced M2-like polarization in LPS-stimulated macrophages. (**A**) QRT-PCR analysis showing the expression levels of *IL-10*, *IL-13*,* Arg1*, *IL-1β*, *IL-6*, and *TNFα* in LPS-stimulated macrophages treated with MSCs-3IL compared to control conditions. Statistical analysis was performed using one-way ANOVA.*P < 0.05, **P < 0.01, ***P < 0.001. *n* = 3 biological replicates. Error bars represent standard deviations (SDs). (**B**) Flow cytometric analysis depicting the percentage of CD163-positive (M2 marker) and CD80-positive (M1 marker) cells in LPS-stimulated macrophages treated with MSCs-3IL

To assess the effect of MSCs-3IL on the macrophage phenotype, Raw264.7 cells were initially stimulated with 100 ng/mL LPS for 24 h to induce the M1 phenotype, followed by treatment with MSCs or MSCs-3IL supernatant for 48 h. The results demonstrated that LPS significantly increased the expression of the proinflammatory cytokines *IL-1β*, *IL-6*, and *TNFα* in Raw264.7 cells, with no significant change in *IL-10* or *Arg1* expression compared with that in untreated controls, indicating that LPS stimulation was able to successfully polarize Raw264.7 cells to the M1 phenotype. Compared with those in the LPS group, the addition of MSCs mixture slightly increased the expression of *IL-13* and *Arg1* and decreased the expression of *IL-1β*, *IL-6* and *TNFα*. Moreover, the MSCs-3IL supernatant significantly increased the expression of *IL-13*, *IL-10*, and *Arg1* and significantly decreased the expression of *IL-1β*, *IL-6*, and *TNFα* (Fig. 4A). Indicating that MSCs drove M2 polarization, which was particularly pronounced when the MSCs-3IL shifted from M1 to M2.

To further demonstrate that MSCs-3IL can induce a shift in the macrophage phenotype, the expression of the M1 marker CD80 reached 91% in Raw264.7 cells after LPS stimulation, as detected by flow cytometry. This expression decreased to 79.9% with the MSCs supernatant and further decreased to 66% with the MSCs-3IL supernatant. MSCs-3IL significantly increased M2 marker CD163 expression to 19%, whereas the MSCs group presented 6% CD163 expression (Fig. 4B). These findings demonstrate that MSCs-3IL effectively promote macrophage polarization toward the M2 phenotype, which contributes to the inhibition of inflammation.

### Migratory response of MSCs to inflammatory and implantational stimuli

**Fig. 5:**
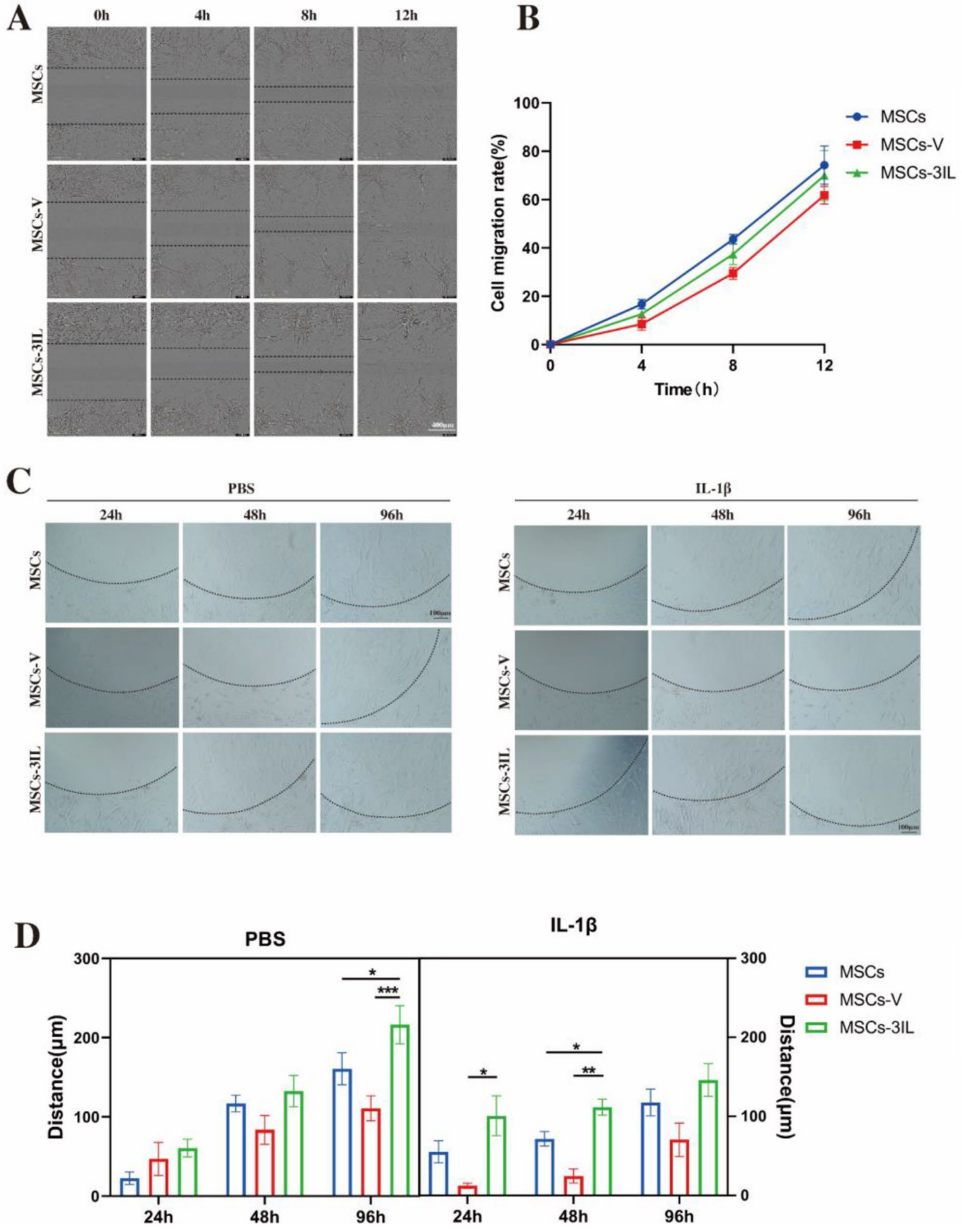
Migration analysis of MSCs under different conditions. (**A**) Cell migration was examined via wound healing assays, in which cells were wounded by scratch injury (black lines), scale bar: 400 μm. (**B**) The cell migration rates were determined. (**C**) Migration analysis in an agarose spot assay, representative images of MSCs migration assay into a PBS-, IL-1β-containing agarose spot at the indicated times, black lines indicate agarose gel boundaries, scale bar: 100 μm. (**D**) The diatance migrated from the border of the anarose spot was measured for MSCs, MSCs-V and MSCs-3IL at the indicated times. Data are presented as mean ± SD. *P < 0.05, **P < 0.01, ***P < 0.001

The migratory ability of different cells was detected by cell scratch assay. According to the results of the wound healing width, the cells of each group were basically under contract after 12 h of scratching, and there was no significant difference in the migration ability between the three groups, indicating that the infection of MSCs-3IL with the three anti-inflammatory factors had no effect on the migration ability of the cells(Fig. 5A and B).

The invasive capacity of all MSCs lines was assessed using the agarose dot assay, which measures the invasive capacity of cells by analyzing their crawling under an agarose gel on a flat surface. All MSCs lines demonstrated the ability to migrate in the agarose droplet assay after 24 h. The migration distance of MSCs-3IL was greater than that of the other cell lines both under non-stimulated conditions and in the presence of the inflammatory cytokine IL-1β(Fig. 5C and D).

### MSCs-3IL accelerated wound healing in diabetic mice

**Fig. 6:**
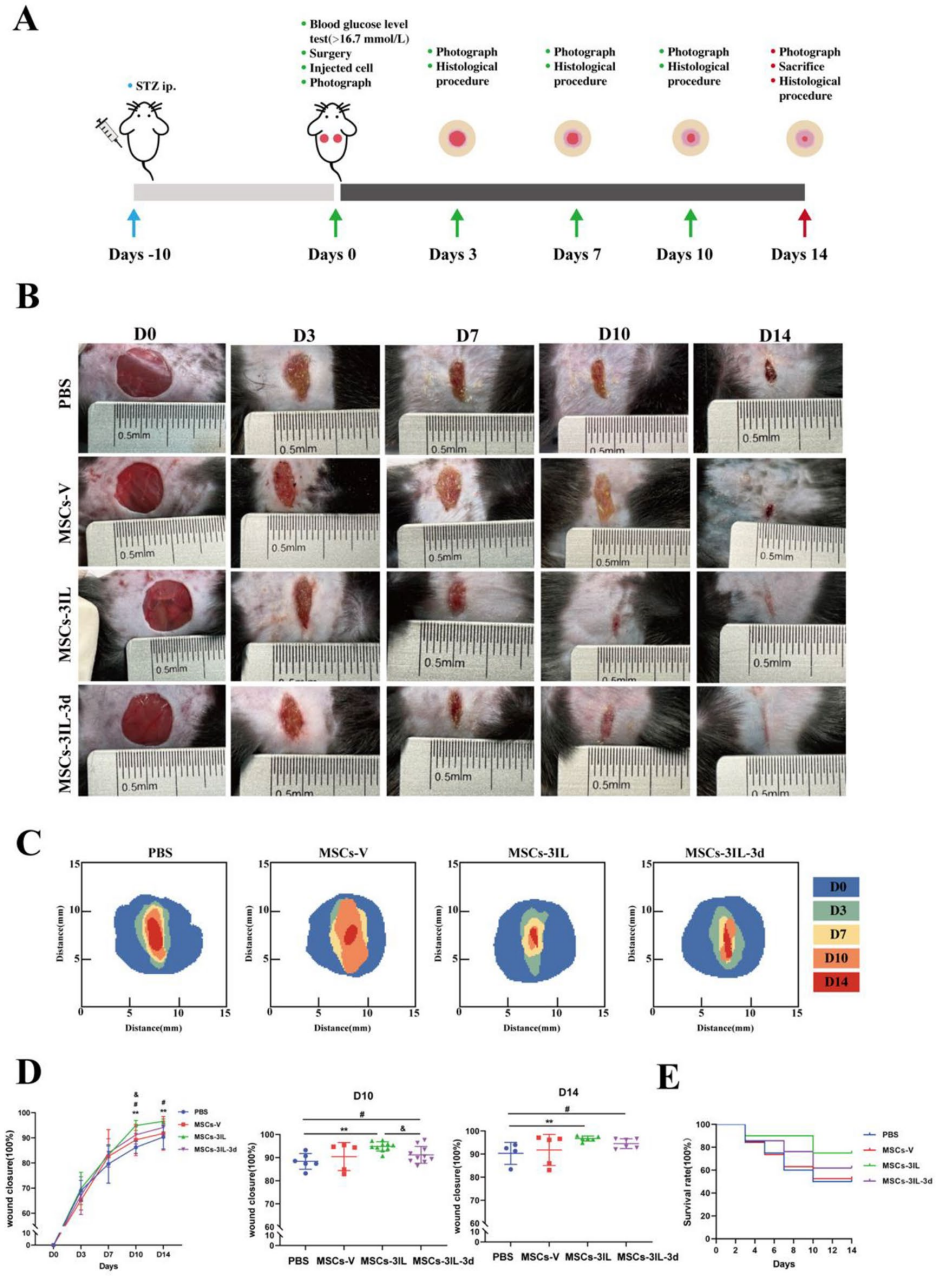
MSCs-3IL accelerated the wound healing of STZ-induced diabetic mice. (**A**) Experimental setup illustrating the creation of a full-thickness skin defect model in STZ-induced diabetic mice and the treatment protocol using PBS, MSCs-V, MSCs-3IL, or MSCs-3IL-3d. (**B**) Representative images illustrating the progression of wound healing in diabetic mice treated with PBS, MSCs-V, MSCs-3IL, or MSCs-3IL-3d over 14 days. (**C**) Schematic diagram illustrating the stages of wound healing and closure over the 14 days. (**D**) Quantification of the wound closure area at different time points (days), with *n* ≥ 5 mice per group. (**E**) Survival rate comparison among the different treatment groups of diabetic mice throughout the experiment

Given the capacity of MSCs-3IL to mitigate inflammation and polarize the macrophage phenotype from M1 to M2 *in vitro*, we wondered whether MSCs-3IL would have the same effect *in vivo* and increase the clinical therapeutic potential. We evaluated the efficacy of MSCs-3IL cell therapy in promoting wound healing in a diabetic mouse model. The establishment and experimental procedure of the mouse diabetic wound model are shown in Fig. 6A. Compared with those in the healthy control group, the fasting blood glucose levels of C57BL/6J mice exceeded 16.7 mmol/L on day 10 post-STZ injection, and the mice in the model group presented with signs of weight loss, polyuria, polydipsia, and polyphagia, confirming successful model induction. To assess the effect of MSCs-3IL, we created two full-thickness 8 mm diameter skin wounds on each mouse’s back, followed by injections categorized into control (PBS), MSCs-V, MSCs-3IL, or MSCs-3ILwhich were administered three days postwound (MSCs-3IL-3d). Wound images were captured at D0, D3, D7, and D14 (Fig. 6B).

The wound closure simulation plots, created from digital images of the healed wounds, revealed that the wounds treated with MSCs-3IL healed the fastest (Fig. 6C). ImageJ analysis of the wound area on D3 and D7 revealed no significant differences among the groups (Fig. 6D). On D10, the wound closure rate was significantly greater in the MSCs-3IL group (94%) and the MSCs-3IL-3d group (91%) than in the PBS group (86%), and the closure rate of the MSCs-V group was 89%. Furthermore, the MSCs-3IL closure rate surpassed that of the MSCs-3IL-3d, suggesting that there was no benefit from delaying treatment postwounding (*p* < 0.05). On D14, the closure rates were 96% in the MSCs-3IL group and 94% in the MSCs-3IL-3d group, which were significantly higher than those in the PBS group (90%), with no difference between the MSCs-3IL group and the MSCs-3IL-3d group. The closure rate was 91% in the MSCs-V group (Fig. 6D). Although there was no difference in the closure rate between the MSCs-V and MSCs-3IL groups, the survival rate of mice in the MSCs-3IL group was higher than that of mice in the other groups (Fig. 6E). These results emphasize that MSCs-3IL therapy accelerates wound healing and may improve survival in diabetic mice, highlighting its potential clinical application. The results suggest that delayed administration did not result in better efficacy. Possibly because inflammation occurs in the early stages of wound healing, delayed injection of cells misses the optimal stage for treating inflammation and does not achieve the desired therapeutic effect.

### MSCs-3IL treatment promoted re-epithelialization and collagen regeneration

**Fig. 7:**
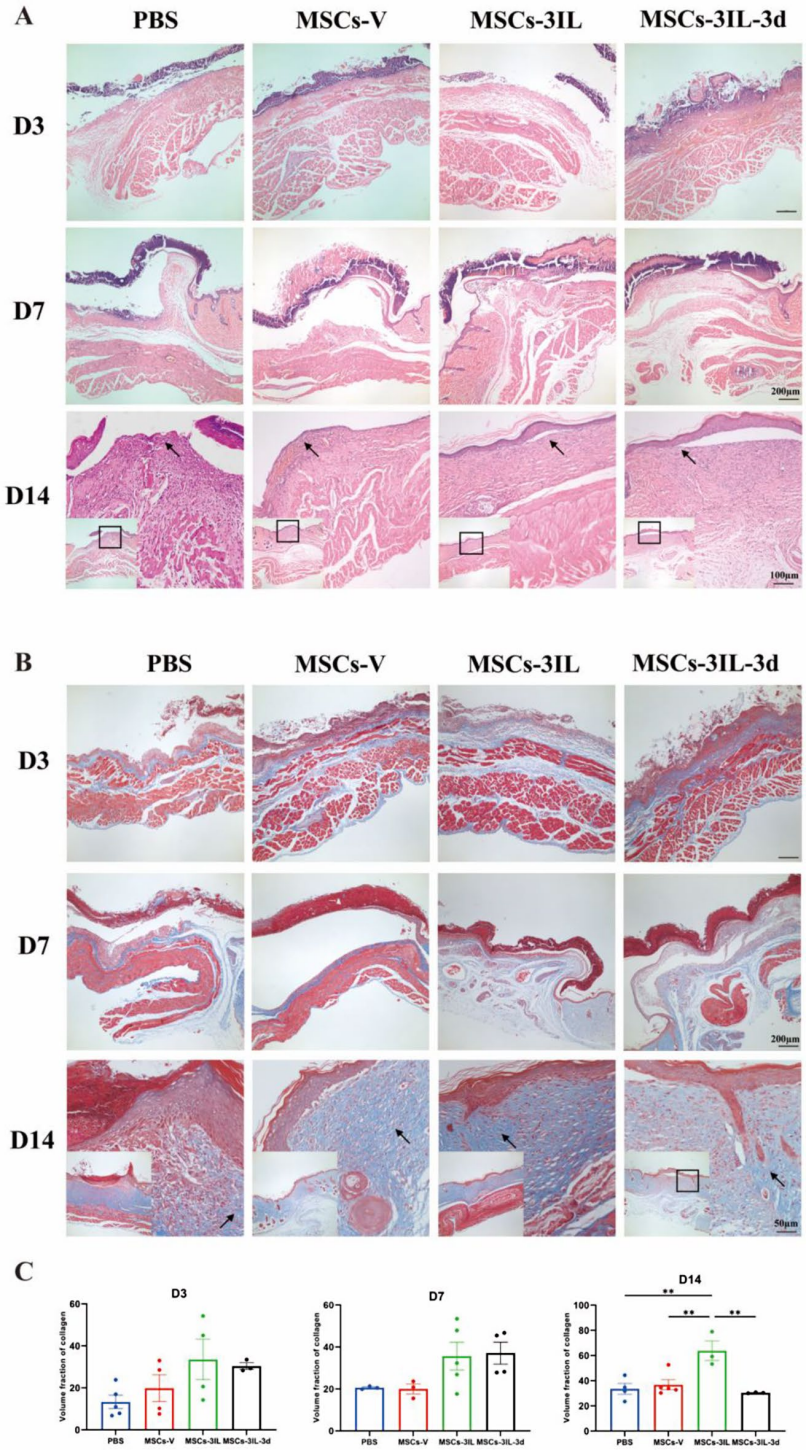
MSCs-3IL treatment promotes angiogenesis and re-epithelialization. (**A**) HE staining of wound sections on D3, D7 and D14 post-operation, arrows indicate regenerating epithelium, scale bar: 100 μm, 200 μm. (**B**) Masson staining of wound sections on D3, D7 and D14 post-operation, arrows point to collagen bundles, scale bar: 100 μm, 200 μm. (**C**) Quantitative analysis of Masson expression in wound tissue on D3, D7 and D14 post-operation. Data are presented as mean ± SD. **P < 0.01

Re-epithelialization in diabetic mouse wounds was assessed via HE staining (Fig. 7A). Significant infiltration of inflammatory cells was observed across all groups on D3. On D7, epidermal defects were observed in the PBS group, and the wounds were infiltrated with many inflammatory cells, whereas the number of inflammatory cells was reduced in the MSCs-V, MSCs-3IL, and MSCs-3IL-3d groups. The MSCs-3IL and MSCs-3IL-3d groups presented a thin epidermal layer and sparse capillaries. On D14, the PBS group still presented a deficient epidermis with persistent inflammatory cell infiltration, whereas the MSCs-V group presented a decreased number of inflammatory cells and the formation of a thin epidermal layer. In contrast, the MSCs-3IL and MSCs-3IL-3d groups presented minimal inflammatory cell infiltration, thicker epidermal layers, and more complete wound epithelia with increased formation of skin appendages, including hair follicles.

The abundance of collagen at the wound site was assessed via Masson’s trichrome staining (Fig. 7B and C). The staining revealed greater collagen formation in the MSCs-V, MSCs-3IL, and MSCs-3IL-3d groups than in the PBS group. On D14, coarse collagen bundles parallel to the surface were evident in the MSCs-3IL and MSCs-3IL-3d groups, compared with the other groups, collagen regeneration was significantly increased in the MSCs-3IL group. Combined, the HE and Masson’s trichrome staining results indicated that MSCs-3IL treatment promoted angiogenesis, improved dermal tissue regeneration, and enhanced collagen regeneration in diabetic wounds, thereby facilitating healing under normal skin conditions.

### MSCs-3IL promoted wound healing by targeting the M2 phenotype in vivo

**Fig. 8:**
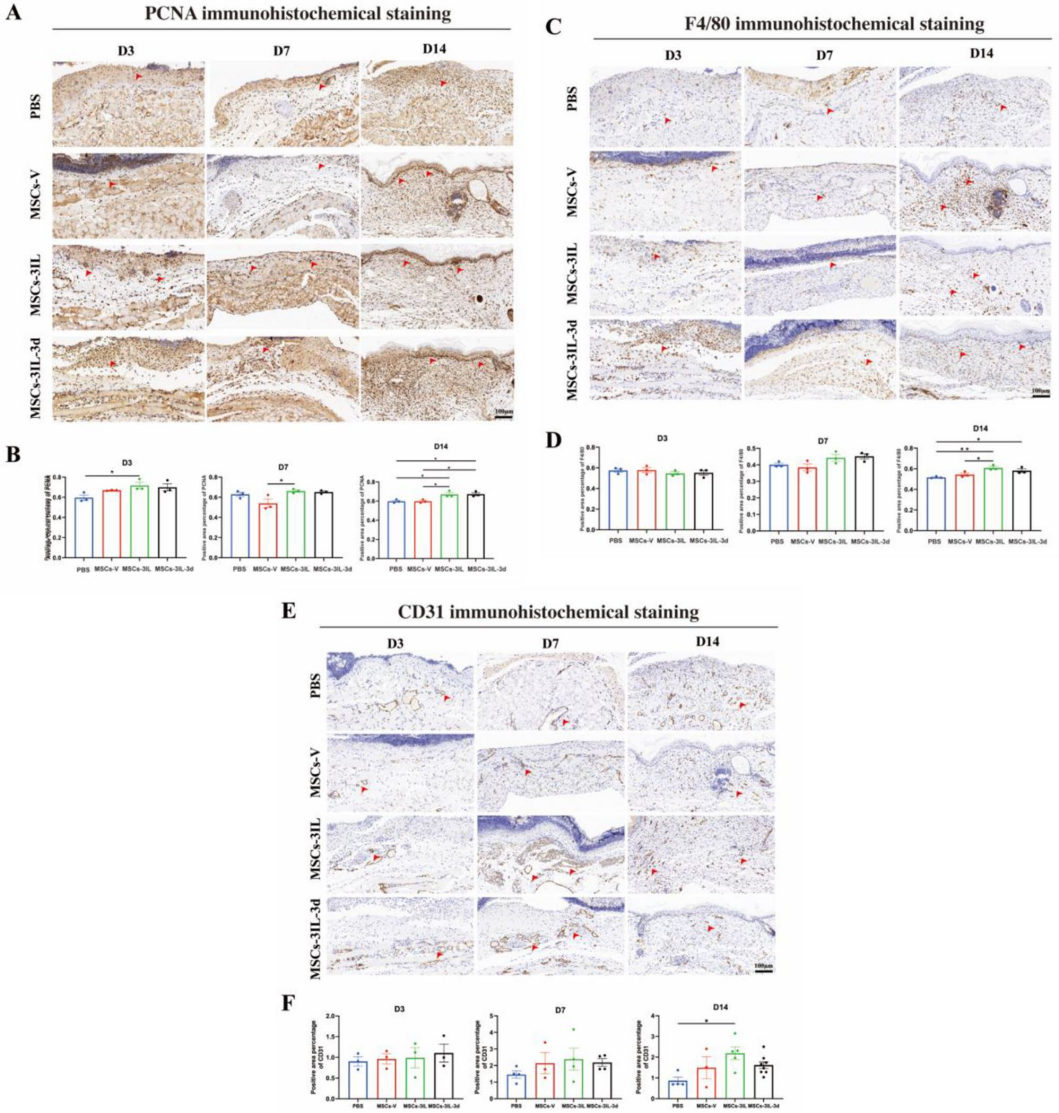
Representative photomicrographs of PCNA, F4/80 and CD31 immune stained sections of the different experimental groups. (**A**) Immunohistochemical staining for PCNA expression in wound tissue on D3, D7 and D14 post-operation, scale bar: 100 μm. (**B**) Quantitative analysis of PCNA expression in wound tissue on D3, D7 and D14 post-operation. (**C**) Immunohistochemical staining for F4/80 expression in wound tissue on D3, D7 and D14 post-operation, scale bar: 100 μm. (**D**) Quantitative analysis of F4/80 expression in wound tissue on D3, D7 and D14 post-operation. (**E**) Immunohistochemical staining for CD31 expression in wound tissue on D3, D7 and D14 post-operation, scale bar: 100 μm. (**F**) Quantitative analysis of CD31 expression in wound tissue on D3, D7 and D14 post-operation. Arrows indicate positive expression. Data are presented as mean ± SD. *P < 0.05, **P < 0.01

To investigate the role of MSCs in wound healing through macrophage modulation, we performed immunohistochemical staining of skin tissues for the proliferation marker PCNA, the macrophage marker F4/80 and the vascular marker CD31. Wound re-epithelialization is crucial for restoring skin barrier integrity and relies on the proliferation, migration, and differentiation of epidermal cells throughout the healing process. Statistical analysis revealed significantly greater PCNA expression in the MSCs-3IL group than in the PBS group on D3, and the PCNA expression in the MSCs-3IL group was greater than that in the MSCs-V group on D7. On D14, PCNA expression remained significantly elevated in both the MSCs-3IL and MSCs-3IL-3d groups compared with that in the PBS and MSCs-V groups (*p* < 0.05), with no significant difference between the MSCs-3IL and MSCs-3IL-3d groups (Fig. 8A and B). These findings indicated that MSCs-3IL treatment promoted the proliferation of epidermal cells and wound re-epithelialization. Quantitative analysis of F4/80-positive areas revealed greater rates in the MSCs-3IL and MSCs-3IL-3d groups than in the PBS group on day 14, with the F4/80-positive area in the MSCs-3IL group also being greater than that in the MSCs-V group (*p* < 0.05) (Fig. 8C and D), suggesting increased macrophage recruitment and inflammation regulation by MSCs-3IL. Quantitative analysis of CD31 immunohistochemistry showed that CD31 was higher in the MSCs-3IL group than in the PBS group at day 14 (*p* < 0.05) (Fig. 8E and F), suggesting that MSCs-3IL increased angiogenesis.

**Fig. 9:**
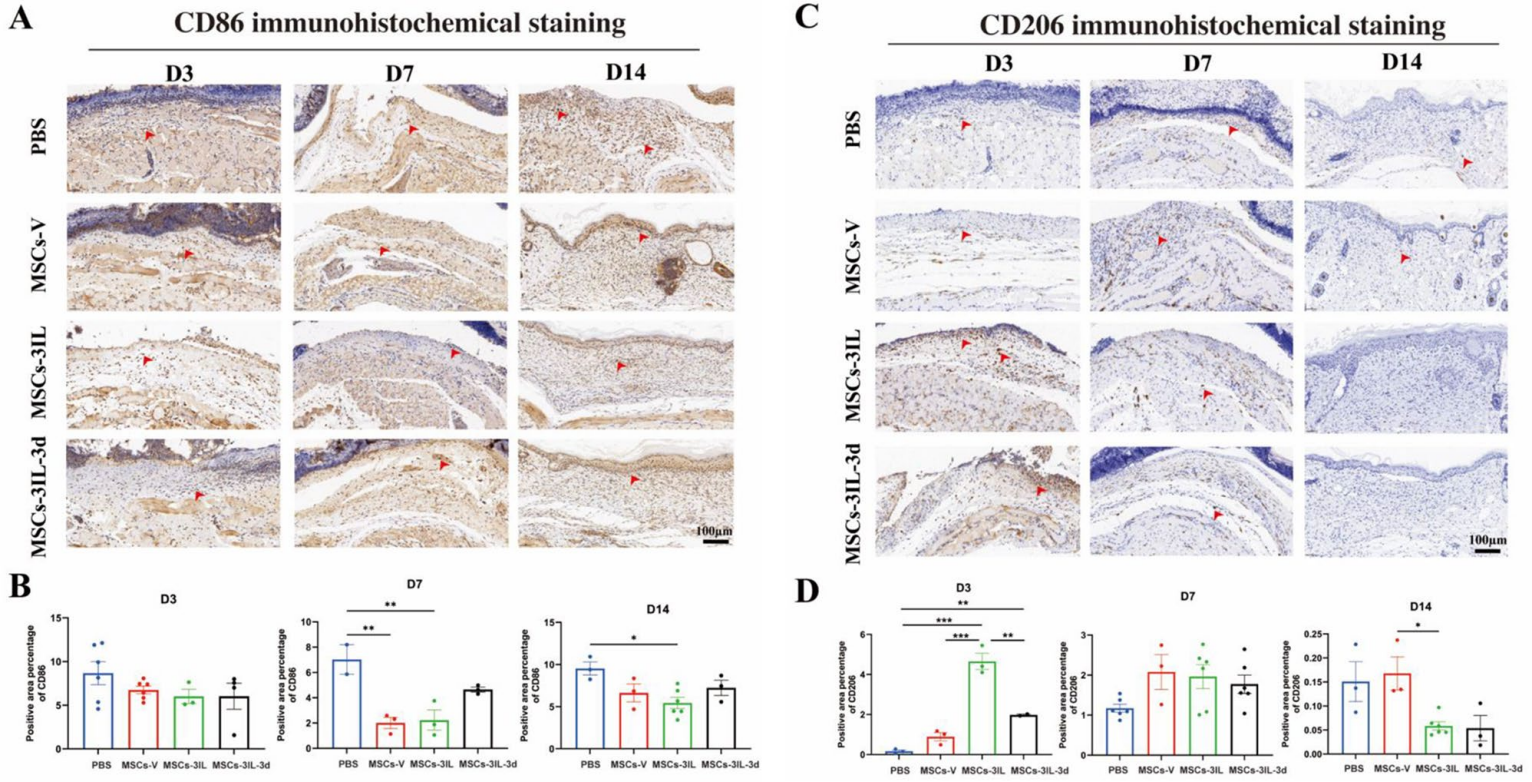
Representative photomicrographs of CD86 and CD206 immune stained sections of the different experimental groups. (**A**) Immunohistochemical staining for CD86 expression in wound tissue on D3, D7 and D14 post-operation, scale bar: 100 μm. (**B**) Quantitative analysis of CD86 expression in wound tissue on D3, D7 and D14 post-operation. (**C**) Immunohistochemical staining for CD206 expression in wound tissue on D3, D7 and D14 post-operation, scale bar: 100 μm. (**D**) Quantitative analysis of CD206 expression in wound tissue on D3, D7 and D14 post-operation. Arrows indicate positive expression. Data are presented as mean ± SD. *P < 0.05, **P < 0.01, ***P < 0.001

To further clarify whether MSCs-3IL promote diabetic wound repair by regulating macrophage phenotype switching, an analysis of CD86 and CD206 immunohistochemical staining results in diabetic wounds revealed that compared with PBS, MSCs-3IL significantly reduced M1 phenotype CD86 expression on D7 and D14, suggesting attenuation of the inflammatory response (Fig. 9A and B). CD206 expression was significantly greater in the MSCs-3IL group than in the control group on day 3, with MSCs-3IL-3d also showing higher expression than PBS. By day 14, CD206 expression in the MSCs-3IL group decreased as wound healing progressed, and the inflammatory response diminished (Fig. 9C and D). In conclusion, MSCs-3IL effectively suppressed the inflammatory response and promoted diabetic wound repair by modulating macrophage phenotype switching, decreasing the M1 proinflammatory phenotype, increasing the M2 anti-inflammatory phenotype, and targeting M2 polarization.

### Changes in local levels of MSCs-3IL cells after injection into diabetic wounds in mice

**Fig. 10:**
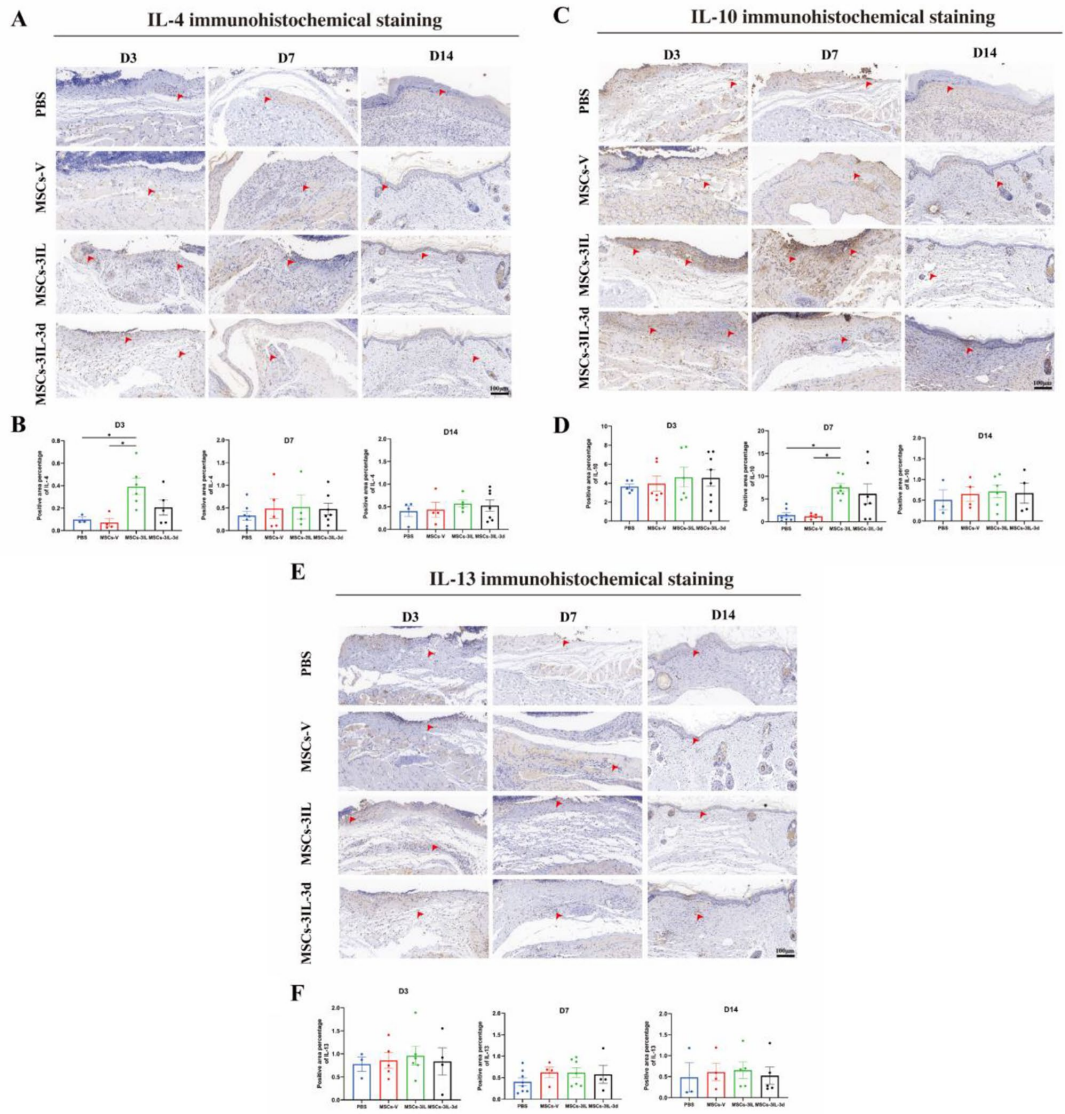
Representative photomicrographs of IL-4, 10–10 and IL-13 immune stained sections of the different experimental groups.(**A**) Immunohistochemical staining for IL-4 expression in wound tissue on D3, D7 and D14 post-operation, scale bar: 100 μm. (**B**) Quantitative analysis of IL-4 expression in wound tissue. (**C**) Immunohistochemical staining for IL-10 expression in wound tissue on D3, D7 and D14 post-operation, scale bar: 100 μm. (**D**) Quantitative analysis of IL-10 expression in wound tissue. (**E**) Immunohistochemical staining for IL-13 expression in wound tissue on D3, D7 and D14 post-operation, scale bar: 100 μm. (**F**) Quantitative analysis of IL-13 expression in wound tissue. Arrows indicate positive expression. Data are presented as mean ± SD. *P < 0.05

After injection of MSCs-3IL into the wounds of diabetic mice, changes in local IL-4, IL-10 and IL-13 concentrations in the wounds of diabetic mice were detected by immunohistochemical results. On D3 after injection, the expression of IL-4 was elevated in the MSCs-3IL group compared to the PBS group and the MSCs-V group. On D7 after injection, the expression of IL-10 was elevated in the MSCs-3IL group compared to the PBS and MSCs-V groups. There was no significant difference in the previous expression of IL-13 between the groups. Changes in these factors occurred mainly within seven days after injection, and none of the differences could be detected at 14 days, suggesting that the effects of the anti-inflammatory factors (IL4, IL-10, and IL-13) were maintained for about seven days (Fig. 10).

### MSCs-3IL treatment in other animal models of inflammation

To further confirm the activity of the genetically modified cells *in vivo*, several other animal models have been developed. In the rat vaginitis model, the degree of redness, swelling of the vaginal opening, and purulent discharge were assessed. Compared with the normal control group, the model group presented severe redness, swelling, and a substantial amount of purulent discharge (Figure S3A). Compared with the model group, the MSCs-V and MSCs-3IL treatment groups presented a marked reduction in vaginal redness, swelling, and purulent discharge, with the MSCs-3IL group exhibiting greater improvement. HE staining revealed that, in the normal control group, the vaginal tissue structure was intact, well-defined, and exhibited normal morphology without inflammatory cell infiltration (red arrows). In the model group, focal degeneration and necrosis of the vaginal mucosal epithelium, proliferation of the lamina propria, and substantial inflammatory cell infiltration in the submucosa were observed (red arrows). Following MSCs-V treatment, minimal degeneration and necrosis of the vaginal mucosal epithelium and slight inflammatory cell infiltration were observed (red arrows). In the MSCs-3IL treatment group, the vaginal morphology appeared relatively intact, without notable inflammatory exudate (red arrows). Overall, compared with MSCs-V, MSCs-3IL had a more pronounced therapeutic effect on the vagina.

In the rat OA model, HE staining revealed severe cartilage destruction, significant inflammatory cell infiltration, and capillary proliferation in the model group (Figure S3B). The MSCs-V treatment group exhibited reduced pathological damage, although inflammatory cell infiltration and capillary proliferation persisted. The MSCs-3IL treatment group demonstrated a significant reduction in pathological damage, with marked improvements in inflammatory cell infiltration and capillary proliferation. Masson staining revealed extensive cartilage destruction in the model group, cartilage regeneration in the MSCs-V treatment group, and cartilage restoration in the MSCs-3IL treatment group compared with the normal control group. These results indicate that, compared with MSCs-V, MSCs-3IL provides superior therapeutic efficacy for OA.

In the mouse acute pleurisy model, HE staining revealed collapsed and atrophied alveoli (blue arrows), thickened bronchial walls (red arrows), and increased inflammatory cell infiltration (green arrows) in the model group (Figure S3C). Following MSCs-V therapy, the alveolar distribution remained uneven (blue arrows), bronchial wall thickness was partially restored (red arrows), and the number of inflammatory cells decreased relative to that in the model group (green arrows). Following MSCs-3IL cell therapy, the alveoli were more evenly distributed, although the morphology was somewhat inferior to that of the normal control group. Compared with those in the MSCs-V treatment group, the bronchial wall thickness was fully restored, and the number of inflammatory cells was further reduced. Overall, compared with MSCs-V, MSCs-3IL had a more pronounced therapeutic effect on lung pleurisy.

HE staining of the mouse acute inflammation model revealed that the lung tissue in the model group exhibited disordered lung tissue structure, with some alveolar cavities atrophied and collapsed (blue arrows), visible bullae, thickened bronchial walls (red arrows), and significant infiltration of inflammatory cells (indicated by the box) (Figure S4A). In the MSCs-V treatment group, the alveoli remained unevenly distributed (blue arrows), the alveolar septa and bronchial walls were thickened (red arrows), and inflammatory cell infiltration persisted (indicated by the box). Compared with the MSCs-V treatment group, the MSCs-3IL treatment group demonstrated significantly better recovery of alveolar structure, with reduced inflammatory cell infiltration in the bronchial walls and alveolar septa, which approached that of the normal lung tissue of the control group. HE staining of the spleen in the normal control group revealed a normal capsule, trabeculae, red pulp, and white pulp structures. In the model group, the white pulp was markedly atrophied, reduced in size, and displayed diffuse disintegration (red arrows), with extensive hemorrhage in the red pulp and poorly defined cellular boundaries. In the MSCs-V treatment group, white pulp atrophy and red pulp hemorrhage were somewhat alleviated. Compared with that in both the model group and the MSCs-V treatment group, the white pulp in the MSCs-3IL treatment group appeared fuller, larger, and overall more normal. A comparison of the effects of different MSCs on the lungs and spleen of mice with acute inflammation revealed that MSCs-3IL had superior anti-inflammatory therapeutic effects.

HE staining of the acute colitis mouse model revealed that the colon tissue in the normal control group had intact mucosal epithelial cells, a normal crypt structure, and orderly arranged glands (Figure S4B). The model group exhibited severe epithelial cell damage and shedding, gland destruction, ulcers (red arrows), and extensive inflammatory cell infiltration. Compared with the model group, the MSCs-V treatment group presented reduced epithelial cell damage, smaller ulcer areas (red arrows), and decreased inflammatory cell infiltration. In contrast, the MSCs-3IL treatment group exhibited only minimal epithelial cell shedding (red arrows). MSCs-3IL effectively inhibited inflammation with a high level of anti-inflammatory activity, better maintaining structural integrity.

HE staining of the pneumonia model revealed disordered lung tissue structure in the model group, with some alveolar cavities atrophied and collapsed, bullae formation, thickened bronchial walls, congested blood vessels, and substantial inflammatory cell infiltration (Figure S4C). In the MSCs-V treatment group, partial recovery of the lung structure was observed in some lobes, whereas other lobes remained collapsed. Compared with the model group, the MSCs-3IL treatment group presented improved alveolar structure, reduced tracheal congestion, and more extensive tissue repair. These findings suggest that MSCs-3IL are more effective than MSCs-V for the acute treatment of diabetes-related pneumonia and exhibit superior anti-inflammatory effects within 6 h.

The above results indicate that MSCs-3IL can effectively treat and accelerate the healing of various animal models of inflammation, providing a strong basis for subsequent clinical application and expansion.

## Discussion

In previous studies, we focused on repairing wounds and inhibiting inflammation [16, 40–43]. In this study, we simultaneously overexpressed three anti-inflammatory cytokines—IL-4, IL-10, and IL-13—in MSCs to enhance diabetic wound repair. By doing so, we successfully achieved robust expression levels of IL-4 (protein secretion 400 ng/mL), IL-10 (200 ng/mL), and IL-13 (6 ng/mL), which greatly exceeded the reported IL-4 secretion of 22 ng/mL in MSCs-IL-4 cells overexpressing IL-4 alone and IL-10 overexpressing IL-10 alone in hAMSC-IL-10 secretion of 600 pg/mL, and IL-13 secretion of 800 pg/mL in the supernatant of ADSCs-IL-13 overexpressing IL-13 [36, 39, 44]. This optimization not only increased cytokine expression but also increased the potential of MSCs to modulate macrophages and promote diabetic wound regeneration. Compared with unmodified MSCs or MSCs-V, MSCs-3IL demonstrated superior induction of macrophage polarization from the proinflammatory M1 phenotype to the anti-inflammatory M2 phenotype in vitro. In a diabetic mouse model treated with MSCs-3IL, significant improvements in epithelial regeneration, collagen deposition, angiogenesis, survival rates, and wound closure rates exceeding 96% by D14 were observed. MSCs-3IL modulated macrophage expression during wound healing and targeted M2 polarization, promoting tissue repair. No difference in the closure rate was observed between the MSCs-3IL and MSCs-3IL-3d groups, indicating that delayed administration did not yield superior outcomes. These results indicate that the overexpression of IL-4, IL-10, and IL-13 enhances the therapeutic efficacy of MSCs in treating diabetic skin wounds.

In addition, we evaluated the safety of MSCs-3IL and the effects of these cells on several other models of inflammation. The enhancement of function did not change the cell phenotype. The dynamic distribution *in vivo* was normal, and no karyotype variation or tumor risk was observed, confirming the safety and feasibility of MSCs-3IL-based therapy. MSCs-3IL can effectively treat and promote healing in animal models of inflammation, such as vaginitis, osteoarthritis, acute pleurisy, acute inflammation, colitis and pneumonia, providing a solid foundation for subsequent clinical application and promotion.

DFU presents a significant challenge in clinical management, and achieving complete wound healing remains difficult [45]. Skin wound healing involves the stages of hemostasis, inflammation, proliferation and remodeling, with macrophages playing pivotal roles as regulators of inflammation and healing [46]. The transition from the inflammatory M1 phenotype to the reparative M2 phenotype is crucial during the shift from the inflammation stage to the proliferation stage and is essential for optimal wound healing [47]. The levels of inflammatory factors and reactive oxygen species (ROS) in M2 macrophages are decreased, whereas the levels of anti-inflammatory cytokines (e.g., TGF-β, IL-10) are increased [12]. M2 macrophages facilitate wound resolution, enhancing angiogenesis, fibroblast function, and extracellular matrix synthesis [48]. As dysregulated macrophage responses are associated with impaired diabetic wound healing, strategies that target macrophages are being explored as promising new therapeutic approaches.

MSCs can modulate immunity and promote tissue regeneration, thereby facilitating the healing of cutaneous wounds [49]. MSCs interact with the immune system mainly through paracrine signaling, where secreted cytokines modulate macrophage polarization without direct cell-to-cell contact [14, 49]. Research has indicated that the interaction between MSCs and macrophages plays an important role in promoting wound healing [50, 51]. The cytokines IL-4, IL-10, and IL-13 play crucial roles in wound healing. They inhibit inflammation, modulate macrophage phenotypes, and promote angiogenesis at various stages of the healing process. IL-4 preserves tissue integrity and stimulates collagen synthesis [52]. IL-10 is an anti-inflammatory and antifibrotic cytokine that can regulate the inflammatory response and influence the extracellular matrix [53]. IL-13 is a key player in triggering inflammation and tissue remodeling, encouraging fibroblast proliferation and differentiation [54]. IL-4 and IL-13 are involved in the inflammatory process of allergic reactions by regulating Th2 cell differentiation and stimulating IgE production by B cells, while IL-10 inhibits Th1 cell activation and proliferation, shifting the immune response to the Th2 type [52, 55]. The three cooperate with each other at different stages of inflammation to form a precise immunoregulatory network and maintain body homeostasis. In macrophages, IL-4 and IL-13 activate the STAT6 pathway via the IL-4 receptor alpha subunit (IL-4Rα), promoting M2 macrophage activation and supporting tissue repair processes [56–58]. IL-10 plays a consolidated anti-inflammatory role in this process by inhibiting pro-inflammatory signaling pathways such as NF-κB in macrophages, further reducing the production of pro-inflammatory cytokines [59]. In conclusion, the three cytokines can synergize their anti-inflammatory and tissue repair functions and regulate macrophage phenotype at different stages of the healing process [58, 60, 61]. Previous gene therapy research has focused on a single specific cytokine, growth factor or chemokine. However, tissue regeneration requires multiple interactions of multiple cells through a large number of cytokines, growth factors or chemokines. Research has indicated that the coexpression of two interacting genes (BMP-4 and hPTH 1–34) accelerates bone formation compared with the use of a single factor alone [62]. Compared with MSCs overexpressing IL-4 alone, those co-overexpressing with PDGF-BB and IL-4 have been shown to increase cell proliferation, activity, and osteogenic capacity [39]. These findings highlight the potential benefits of synergistic cytokine expression over individual modifications.

Our findings highlight that MSCs-3IL therapy effectively promotes diabetic wound healing by reducing M1 polarization and enhancing M2 polarization. In addition to the mechanism by which MSCs-3IL can treat diabetic foot ulcers by inducing M2 polarization, MSCs-3IL also promotes vascular regeneration and tissue damage repair. Increasing evidence suggests that MSCs can promote local blood flow reconstruction by paracrine secretion of multiple pro-angiogenic factors, promoting extracellular matrix production and reducing endothelial cell apoptosis, which plays an important role in angiogenesis in many pathological processes (e.g., wound healing, bone repair, and myocardial infarction) [63–65]. Some studies have used hUCMSCs to induce β-catenin activation in endothelial cells by inducing YAP phosphorylation affecting the Wnt4 signaling pathway to promote angiogenesis [66]. Meanwhile, MSCs can differentiate into endothelial cells and smooth muscle cells of blood vessels and promote vascular regeneration [67].

In addition, many basic and clinical studies have demonstrated that MSCs have desirable effects on tissue repair of chronic refractory wounds in diabetic patients [13, 68]. In our mouse diabetic wound model, MSCs-3IL effectively promoted wound healing and accelerated wound repair. As seen by HE and Masson staining results, the skin of mice in the MSCs-3IL group exhibited minimal inflammatory cell infiltration, a thicker epidermal layer and a more intact skin epithelium, and enhanced collagen regeneration at the wound site. When we tested delayed administration treatment, we found that it did not produce better outcomes than what was reported in the literature [69]. This may be related to the fact that inflammation occurs in the early stages of wound healing, and the wound transitions to M2 macrophages around day three after injury. Therefore, injecting cells three days later may have missed the stage of inflammation that occurs [12, 31]. Future investigations should focus on elucidating the precise mechanisms by which IL-4, IL-10, and IL-13 orchestrate wound healing through M2 polarization and other pathways, which could open the door for clinical applications of MSCs-3IL in enhancing wound repair and regeneration.

## Conclusions

This study introduces a novel approach for treating diabetic wounds via cell therapy. This approach involves modifying hUMSCs to simultaneously overexpress multiple factors. By introducing the IL-4, IL-10, and IL-13 genes into hUMSCs, the resulting MSCs-3IL exhibited strong expression of anti-inflammatory factors and improved wound healing and promoted M2 polarization compared with unmodified hUMSCs. This strategy demonstrates that targeting M2 polarization is a promising alternative for DFU treatment. Importantly, the study revealed no chromosomal mutations or tumorigenic outcomes, confirming the safety and feasibility of cell-based therapy.

## Electronic supplementary material

Below is the link to the electronic supplementary material.

Supplementary Material 1

Supplementary Material 2

## Data Availability

The data that support the findings of this study are available within this article or from the corresponding author upon reasonable request.
