# Supplementary material for: Preclinical study of engineering MSCs promoting diabetic wound healing and other inflammatory diseases through M2 polarization
Source: Stem Cell Res Ther. 2025 Mar 5;16:113. doi: 10.1186/s13287-025-04248-y (PMC11881511; doi:10.1186/s13287-025-04248-y)
Supplement: Supplementary file 1 — Supplementary Material 1 [file 13287_2025_4248_MOESM1_ESM.docx]

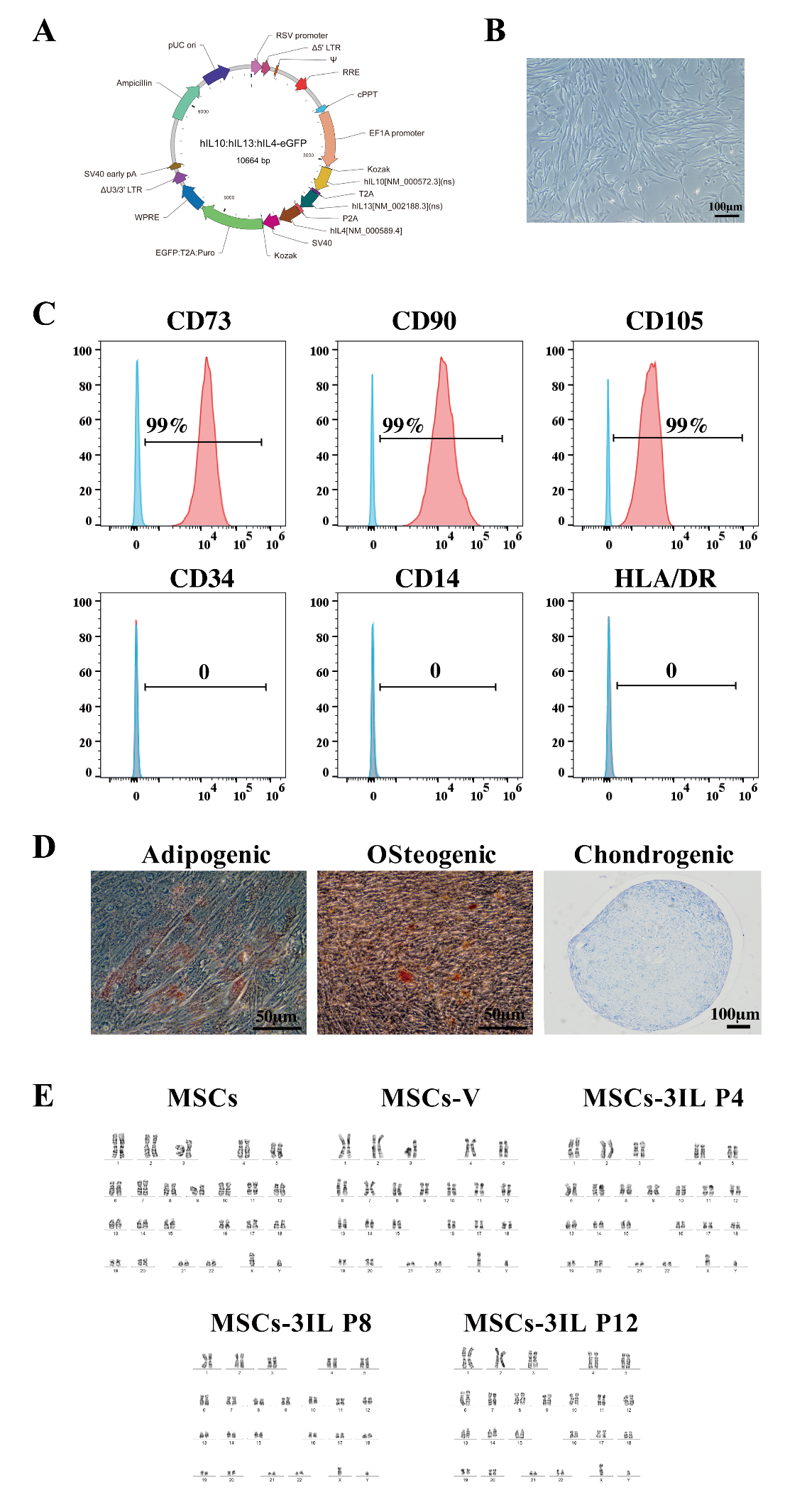


**Supplementary Figure 1. Characterization of MSCs and MSCs-3IL.** (A) Lentiviral vector map illustrating the overexpression of IL-4, IL-10, and IL-13 genes in MSCs-3IL. (B) Microscopy image of cultured MSCs, scale bar:100 μm. (C) Flow cytometry analysis of MSCs surface markers: CD73, CD90, CD105 (positive markers); CD34, CD14, HLA-DR (negative markers). (D) Adipogenic, osteogenic, and chondrogenic differentiated MSCs were stained with alizarin red S, oil red O, and alizarin blue, scale bar: 50μm, 100 μm. (E)Karyotype analysis of MSCs, MSCs-V, MSCs-3IL.


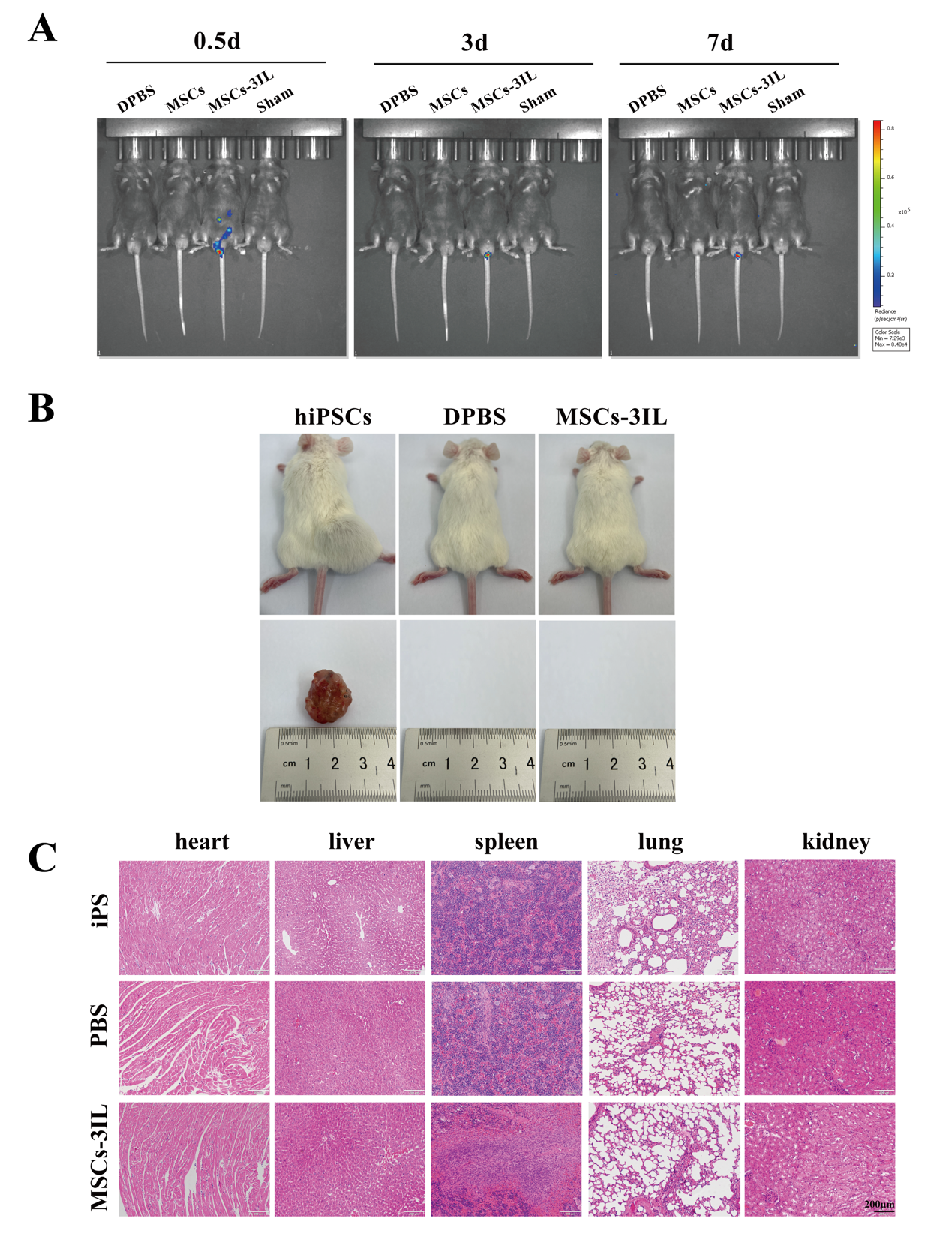


**Supplementary Figure 2. Verification of the safety of MSCs virus infection.** (A) Image of luciferase-expressing MSCs *in vivo* of the mice. (B) Tumor formation assay comparing hiPSCs, PBS, and MSCs-3IL, evaluating the potential tumorigenicity associated with MSCs after virus infection. (C) HE staining of heart, liver, spleen, lung and kidney tissues taken from the hiPSCs, PBS and MSCs-3IL groups in the tumor formation assay, scale bar: 200 μm.


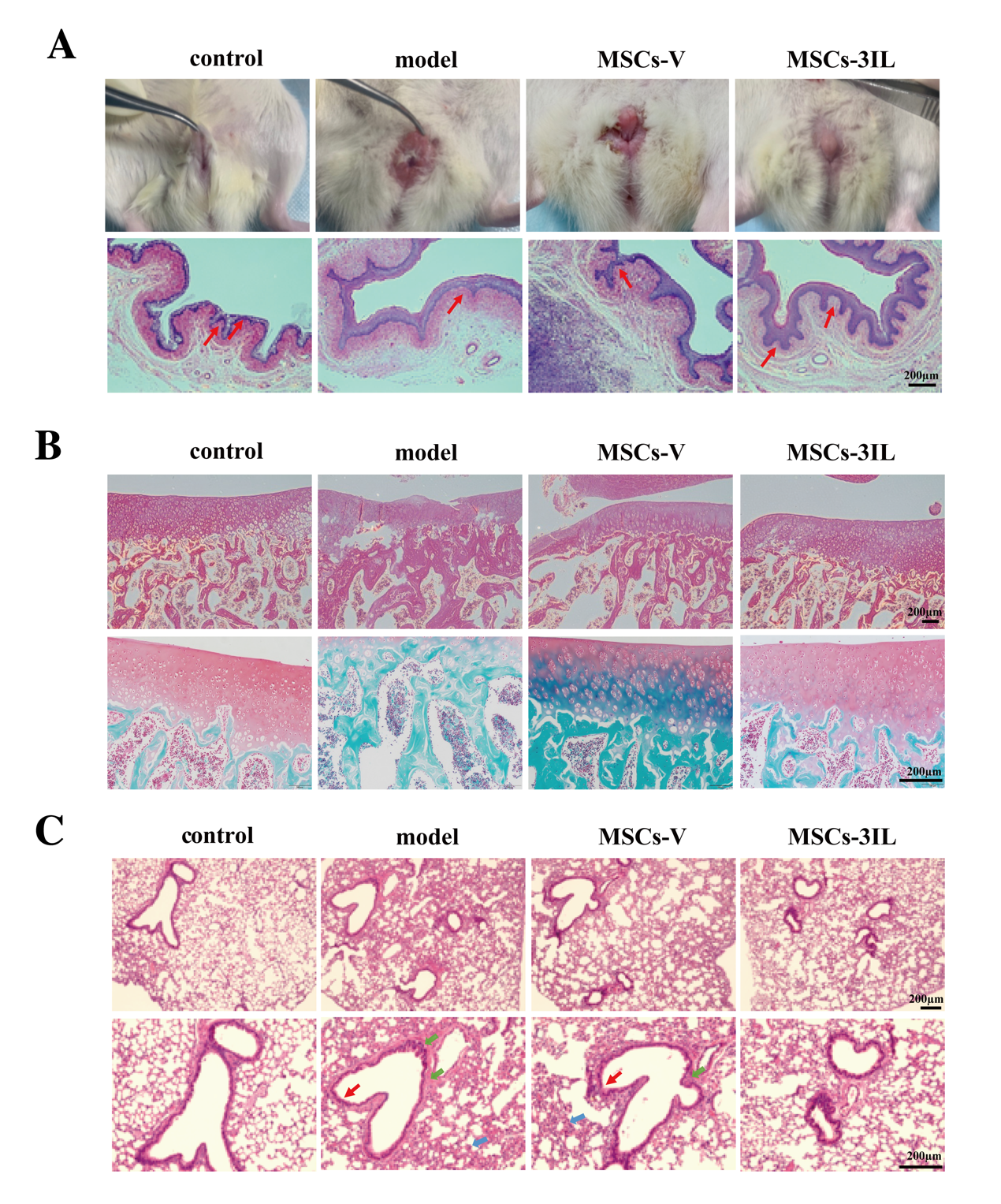


**Supplementary Figure 3. MSCs-3IL treatment in animal models of vaginitis, osteoarthritis and acute pleurisy.** (A) Cell therapy in the rat vaginitis model, scale bar: 200 μm. (B) Cell therapy in the rat osteoarthritis model, scale bar: 200 μm. (C) Cell therapy in the mouse acute pleurisy model, scale bar: 200 μm.


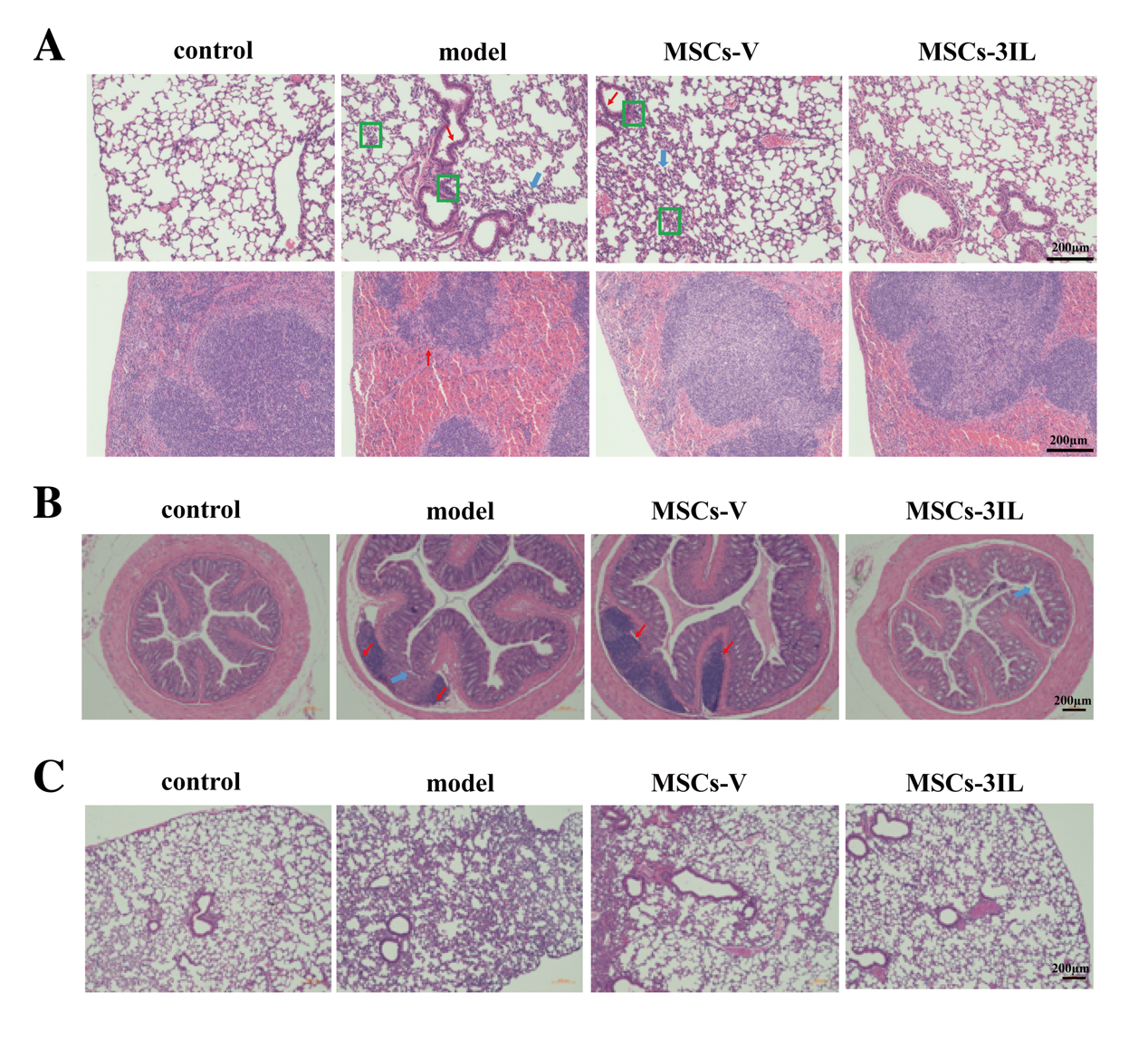


**Supplementary Figure 4. MSCs-3IL treatment in animal models of acute inflammation, colitis, and pneumonia.** (A) Cell therapy in the mouse acute inflammation model, scale bar: 200 μm. (B) Cell therapy in the mouse colitis model, scale bar: 200 μm. (C) Cell therapy in the mouse pneumonia model, scale bar: 200 μm.
