# Supplementary material for: Preclinical study of engineering MSCs promoting diabetic wound healing and other inflammatory diseases through M2 polarization
Source: Stem Cell Res Ther. 2025 Mar 5;16:113. doi: 10.1186/s13287-025-04248-y (PMC11881511; doi:10.1186/s13287-025-04248-y)
Supplement: Supplementary file 2 — Supplementary Material 2 [file 13287_2025_4248_MOESM2_ESM.docx]

**
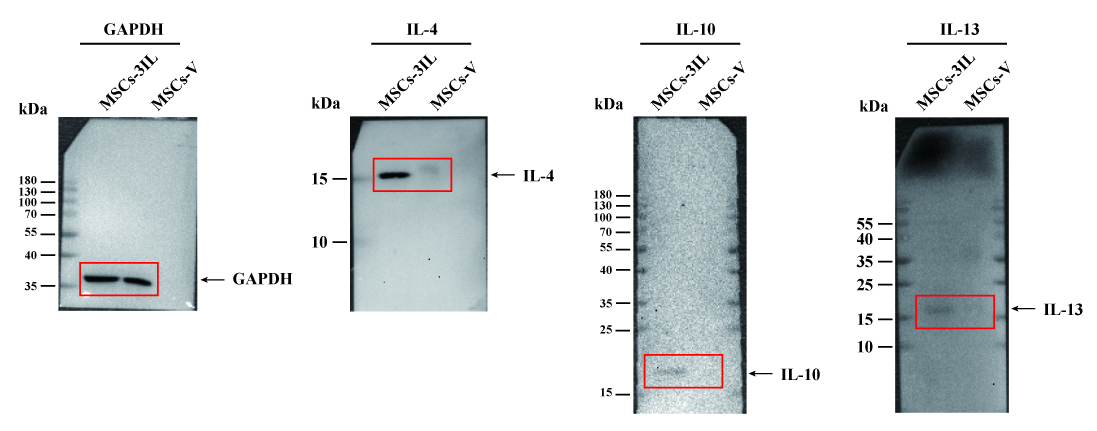
**

**Supplementary material. Western blot analysis showing protein expression levels of IL-4, IL-10, and IL-13 in MSCs-3IL and MSCs-V.** As the original full-length uncropped blots of IL-4, IL-10 and IL-13 protein expression levels in MSCs-3IL and MSCs-V in Figure 3(D), red boxes are the blots shown in Figure 3(D).
